# Multiscale adaptive differential abundance analysis in microbial compositional data

**DOI:** 10.1093/bioinformatics/btad178

**Published:** 2023-04-05

**Authors:** Shulei Wang

**Affiliations:** Department of Statistics, University of Illinois at Urbana-Champaign, Champaign, IL 61820, USA

## Abstract

**Motivation:**

Differential abundance analysis is an essential and commonly used tool to characterize the difference between microbial communities. However, identifying differentially abundant microbes remains a challenging problem because the observed microbiome data are inherently compositional, excessive sparse, and distorted by experimental bias. Besides these major challenges, the results of differential abundance analysis also depend largely on the choice of analysis unit, adding another practical complexity to this already complicated problem.

**Results:**

In this work, we introduce a new differential abundance test called the MsRDB test, which embeds the sequences into a metric space and integrates a multiscale adaptive strategy for utilizing spatial structure to identify differentially abundant microbes. Compared with existing methods, the MsRDB test can detect differentially abundant microbes at the finest resolution offered by data and provide adequate detection power while being robust to zero counts, compositional effect, and experimental bias in the microbial compositional dataset. Applications to both simulated and real microbial compositional datasets demonstrate the usefulness of the MsRDB test.

**Availability and implementation:**

All analyses can be found under https://github.com/lakerwsl/MsRDB-Manuscript-Code.

## 1 Introduction

Differential abundance analysis is a commonly used tool to decipher the difference between microbial communities and identify dysbiotic microbes. The primary goal of differential abundance analysis is to detect a set of microbes associated with experimental conditions based on observed count data generated from high-throughput sequencing technologies. Although popular, differential abundance analysis in microbiome data remains a challenging statistical problem as the observed count data are inherently compositional (Li 2015; Gloor et al. 2017; Vandeputte et al. 2017; Morton et al. 2019), excessive sparse (Brill et al. 2022), and distorted by experimental bias (McLaren et al. 2019) (Supplementary Fig. S1). To address these challenges, a number of studies have proposed differential abundance analysis procedures for microbiome data (Fernandes et al. 2013; Paulson et al. 2013; Mandal et al. 2015; Shi et al. 2016; Tang et al. 2017; Xiao et al. 2017; Chen et al. 2018; Morton et al. 2019; Martin et al. 2020; Lin and Peddada 2020a; Zhou et al. 2021; Brill et al. 2022; Zhou et al. 2022; Wang 2023). See a comprehensive review of differential abundance analysis in Weiss et al. (2017) or Lin and Peddada (2020b).

Before applying these existing methods, one must choose an appropriate analysis unit for differential abundance analysis. The reads in 16S rRNA sequencing data are clustered into operational taxonomic units (OTUs) (Hamady and Knight 2009) or denoised as amplicon sequence variants (ASVs) (Callahan et al. 2016; Amir et al. 2017). Using OTUs or ASVs as the analysis unit, we can detect the differentially abundant microbes at the finest resolution offered by 16S rRNA sequencing data (Fig. 2A). However, such a strategy sometimes can suffer a loss of detection power as the sparse counts in each OTU or ASV are not informative enough for an effective comparison (Huang et al. 2021). To increase power, one commonly used strategy is to aggregate similar OTUs or ASVs as taxa, such as genera, according to their assigned/mapped taxonomy (Fig. 2B). Treating taxa as analysis units can provide more power to detect differentially abundant microbes but at a coarser resolution. Choosing a taxonomy rank in the taxon-wise analysis also needs facing a tradeoff between resolution and sequence utilization. On the one hand, a lower taxonomy rank can provide finer resolution but requires throwing out many unassigned sequences due to the current coarse taxonomic classification. On the other hand, a higher taxonomy rank can keep and utilize more sequences but loses resolution to capture the heterogeneous signal. Besides aggregating based on assigned taxonomy, another strategy to combine the OTUs or ASVs is to incorporate the hierarchical structure of the phylogenetic tree (Yekutieli 2008; Tang et al. 2017; Xiao et al. 2017; Heller et al. 2018; Bichat et al. 2020; Huang et al. 2021; Li et al. 2022). Such a strategy allows detection of the differential abundant microbes at different resolutions and improves the detection power compared with OTU/ASV-wise analysis. However, this strategy usually requires accurate evolution information of the phylogenetic tree, and it is still unknown how the error in phylogenetic tree construction affects the downstream differential abundance analysis. It is natural to wonder if there is a way to detect differentially abundant microbes at OTU or ASV resolution robustly while maintaining enough power and using all available sequences. Motivated by this need, we introduce an alternative way to conduct differential abundance analysis in this article.

Here, we propose the MsRDB test (Multiscale Adaptive Robust Differential Abundance Test), a multiscale method for differential abundance analysis in microbial compositional data. Instead of independent units, the MsRDB test embeds ASVs’ sequences or OTUs’ representative sequences into a metric space where their pairwise distance can be utilized. The metric space embedding allows us to utilize their spatial structure to gain more detection power. To identify multiscale signals, the MsRDB test adopts a propagation and separation (PS) approach (Polzehl and Spokoiny 2000, 2006) to aggregate information from the neighborhood iteratively and adaptively. Notably, the MsRDB test only integrates OTUs/ASVs with similar levels of differential abundance so that we can capture the heterogeneous signal easily and avoid false discovery inflation caused by aggregation. As a generalization of robust differential abundance (RDB) test (Wang 2023), the MsRDB test is also an iterative empirical Bayes method and thus inherits many good properties from the RDB test, including robustness to zero counts, compositional effect, and experimental bias. We evaluate the performance of the MsRDB test using both simulated and 16S rRNA dataset from published studies. Through these numerical examples, we compare the MsRDB test with ASV-wise and taxon-wise analysis. We demonstrate that the MsRDB test can help reveal the multiscale differentially abundant microbes at ASV resolution, providing more insights into the subtle difference between microbial communities.

## 2 Materials and methods

**Figure 1.**
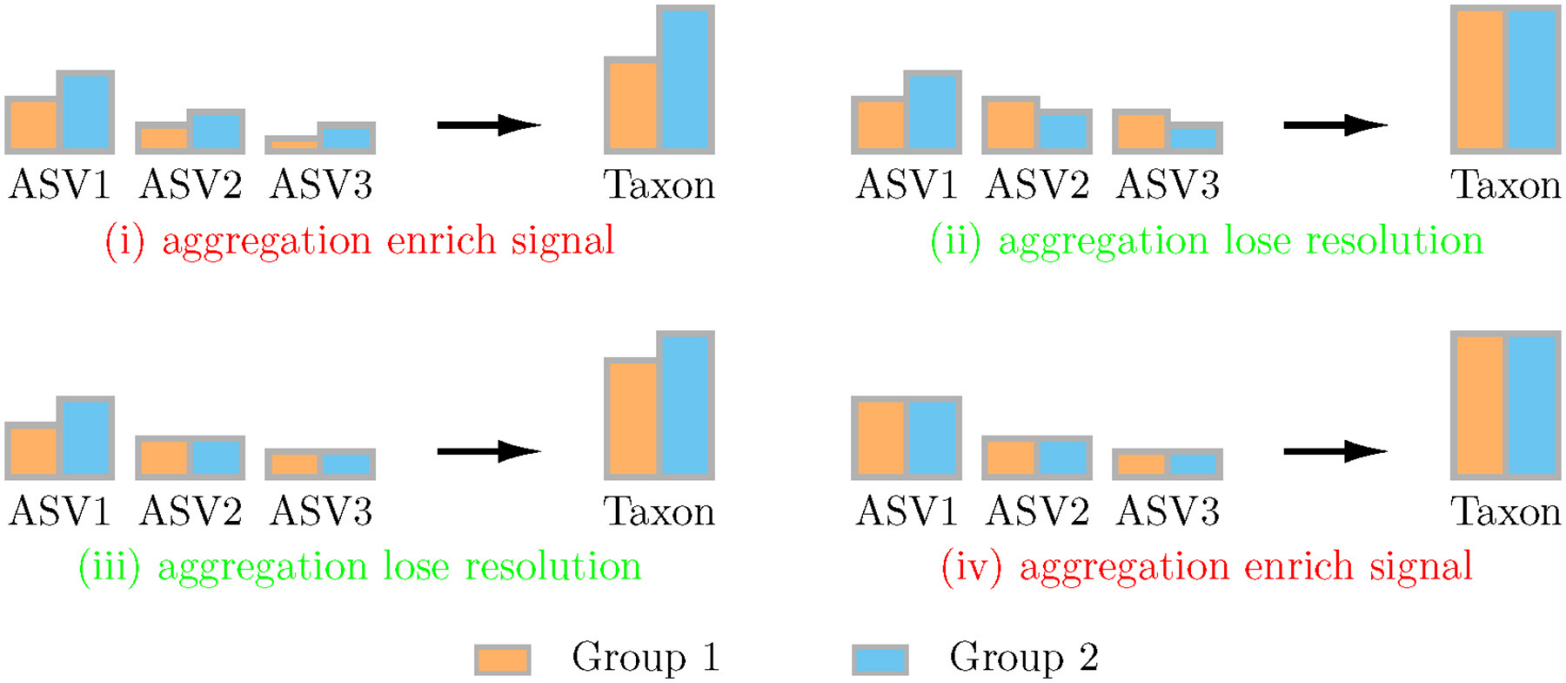
The effect in ASV aggregation. This figure illustrates four possibilities of ASV aggregation: (i) aggregate differential abundant ASVs with a similar level of differential abundance (e.g. all ASVs’ abundance in Group 1 is smaller than abundance in Group 2); (ii) aggregate differential abundant ASVs with different level of differential abundance (e.g. some ASVs’ abundance in Group 1 are smaller than abundance in Group 2 and some are larger); (iii) aggregate differential abundant ASVs and nondifferential abundant ASVs; and (iv) aggregate nondifferential abundant ASVs. The aggregation in (i) and (iv) are beneficial as they can help enrich the signal, while the aggregation in (ii) and (iii) can make the analysis lose resolution and cancel out the signal. The bar plots in this figure represent absolute abundance

Before introducing the new MsRDB test, we first discuss why the results of differential abundance analysis highly rely on the choice of analysis unit. We focus primarily on the ASVs in this work for concreteness and illustration, while all the discussions and techniques introduced here readily apply to OTUs. The main idea in the taxon-wise analysis is to aggregate data of ASVs under the guidance of the assigned taxonomy. The way to aggregate ASV greatly impacts the results of differential abundance analysis. On the one hand, the aggregation usually enriches the differential abundant signal provided the ASVs assigned to the same taxon have similar levels of differential abundance (Fig. 1i and iv). On the other hand, when the ASVs have different levels of differential abundance, aggregation could lead to canceling out differential abundant signal (Fig. 1ii) and losing resolution (Fig. 1iii). However, the current taxonomy-based aggregation has no guarantee to ensure aggregation of ASVs with similar levels of differential abundance and may result in throwing out a considerable amount of unassigned ASVs. Can we design a new way of ASV aggregation that keeps all sequences in the analysis and only aggregate ASVs with similar levels of differential abundance?

**Figure 2.**
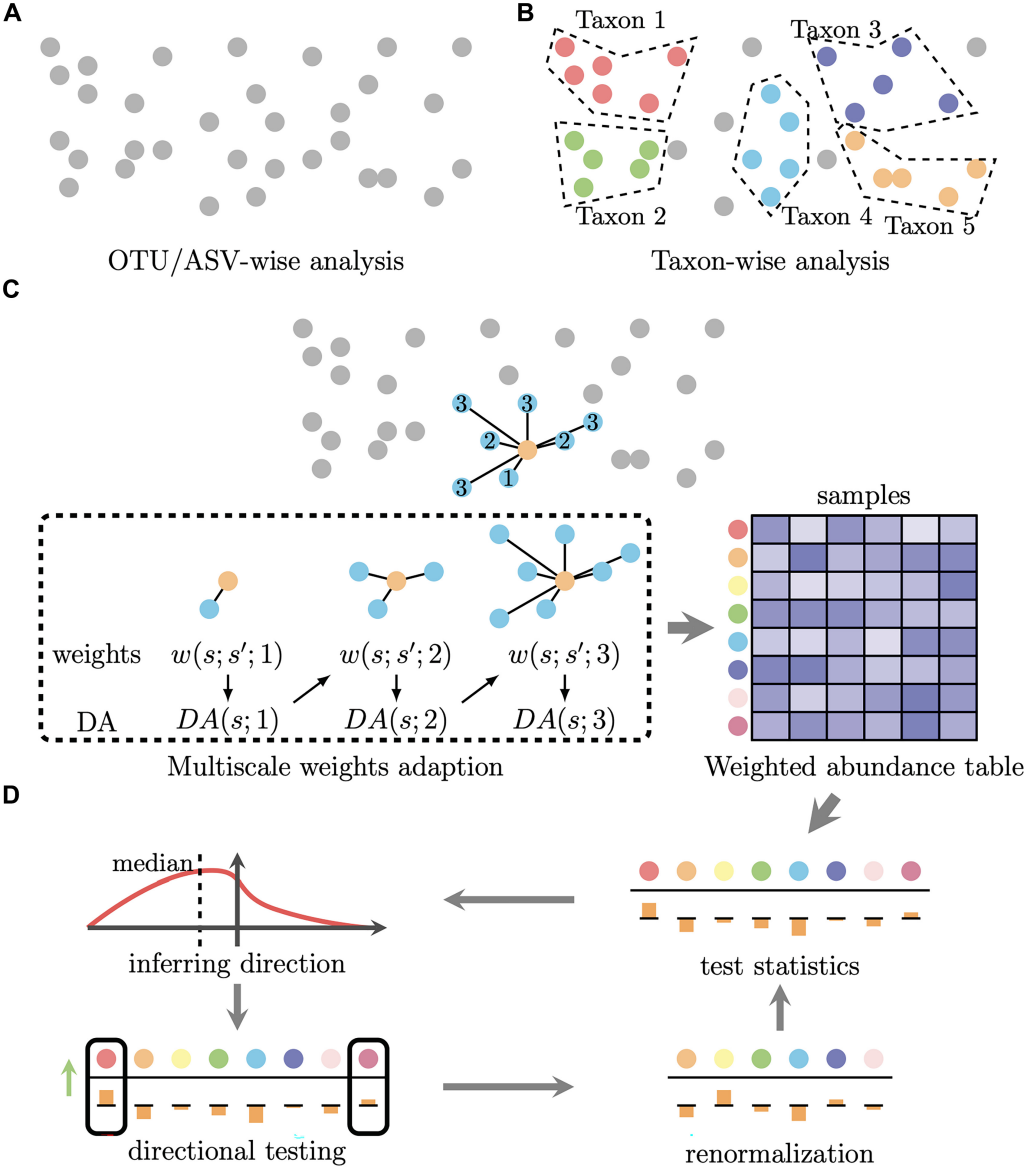
Illustration demonstrating OTU/ASV-wise analysis, taxon-wise analysis, and MsRDB test. (A) OTU/ASV-wise analysis treats OTUs or ASVs as independent units in differential abundance analysis. (B) According to the mapped or assigned taxon, OTUs or ASVs are first grouped as taxa. The taxa are regarded as independent units in taxon-wise analysis. (C) The first step in the MsRDB test constructs a weighted count table by exploiting the spatial structure of ASVs. Instead of independent units, the MsRDB test embeds the ASVs into a metric space and utilizes their spatial structure to gain more power. The PS approach used in the MsRDB test allows borrowing strength from the neighborhood in a multiscale and adaptive way. (D) The second step in the MsRDB test identifies differential ASVs by iterative directional two-sample tests and renormalization

We give a brief overview of the new MsRDB test, and a detailed description is explicated in the Supplementary Material. The MsRDB test consists of two main steps: (i) the ASV aggregation by a multiscale and adaptive method (Fig. 2C) and (ii) differential abundance analysis on the weighted abundance table (Fig. 2D). In the first step (Fig. 2C), the MsRDB test embeds all ASVs’ sequences into a metric space so we can directly measure the similarities between any pair of ASVs’ sequences. Unlike the taxon-wise analysis, which measures ASVs’ similarity by assigned taxonomy, the idea of embedding into a metric space allows the MsRDB test to keep all sequences in the analysis. After embedding, the MsRDB test aggregates the data in each ASV’s neighborhood by assigning weights to each pair of ASVs *s* and $s^{\prime }$


$$w(s;s^{\prime })=K_{s}\left(\frac{d(s,s^{\prime })}{r}\right)K_{a}\left(\frac{D(\hat{DA}(s);\hat{DA}(s^{\prime }))}{h}\right).$$

The weights we use here reflect two types of information: how different the two ASVs’ sequences are and how different the levels of differential abundance are. Here, $K_{s}$ is a kernel used to measure the sequence similarity, and $K_{a}$ is designed to measure the similarity of differential abundance level. The differential abundance level in $w(s;s^{\prime })$ is measured by a robust coefficient of estimated fold change where ${\bar{P}}_{k,w}(s)$ is the weighted mean of the relative abundance of *s* in the *k*th group, i.e. ${\bar{P}}_{k,w}(s)=\sum_{s^{\prime }\in S}w(s;s^{\prime }){\bar{P}}_{k}(s^{\prime })$, where ${\bar{P}}_{k}(s)$ is the mean of the relative abundance of *s* in the *k*th group. Incorporating weights $w(s;s^{\prime })$ in ${\bar{P}}_{k,w}(s)$ allows a more accurate estimation of differential abundance level but also raises a challenge in simultaneously evaluating weights and differential abundance level. Since $w(s;s^{\prime })$ and $\hat{DA}(s)$ rely on each other, we adopt an iterative and multiscale approach, called the propagation and separation method (PS), to update weights and the estimator for differential abundance level simultaneously and adaptively in a series of nested neighborhoods (Fig. 2C). Through the PS approach, the weights in the MsRDB test allow each ASV to effectively borrow strength from other similar ASVs, thus increasing the power in detecting more subtle levels of differential abundance. Importantly, the weights in the MsRDB test reflect similarities of ASVs’ sequences and their differential abundance levels so that only ASVs with similar levels of differential abundance are aggregated. Based on the assigned weights, we aggregate the relative abundance of ASVs to obtain a weighted abundance table, where each row still corresponds to each ASV.


$$\hat{DA}(s)=\frac{{\bar{P}}_{1,w}(s)-{\bar{P}}_{2,w}(s)}{{\bar{P}}_{1,w}(s)+{\bar{P}}_{2,w}(s)},$$

In the second step (Fig. 2D), we conduct differential abundance analysis on the weighted abundance table obtained in the first step. Specifically, we formulate the differential abundance analysis in microbial compositional data as a reference-based hypothesis to account for the compositional nature of data and potential experimental bias (Brill et al. 2022). In the reference-based hypothesis, we do not directly test whether the relative abundance changes across groups, but we need to test if the fold change of each ASV is different from the majority of ASVs. To test such a hypothesis, we introduce a weighted version of the RDB test for the weighted abundance table, a generalized version of the vanilla RDB test (Wang 2023). Like the vanilla RDB test, the weighted version RDB test also exploits the fact that the nondifferential abundant ASVs share the same fold change, so the expectation of two-sample *t*-tests on them have the same signs. Using this fact, the MsRDB test adopts an iterative empirical Bayes framework to identify differential abundant ASVs. More concretely, the iterative empirical Bayes framework consists of two steps in each iteration: (i) integrates two-sample *t*-test statistics to infer the testing direction and then conduct directional two-sample *t*-test; (ii) renormalizes the weighted abundance table with respect to the active ASVs (Fig. 2D). Since only two-sample *t*-test statistics are evaluated, there is no need for an extra step to handle zero counts in the MsRDB test. Compared with the vanilla RDB test, the MsRDB test is designed for a weighted abundance table and uses ASVs’ taxonomy only for results interpretation instead of data aggregation.

In the MsRDB test, we need to specify two sets of tuning parameters: one is for the PS method, and the other is for the weighted RDB test. In the MsRDB test, we choose the tuning parameters of the PS method similar to the literature (Li et al. 2011; Wang et al. 2019). The performance of the MsRDB test is not very sensitive to most tuning parameters. The most sensitive parameter is the number of neighbors in the last iteration *k*. As shown in Section 3.1, the false discovery rate (FDR) may be slightly inflated when the number of neighbors *k* increases. In the weighted RDB test, we can choose the tuning parameters like the original RDB test. In particular, the most sensitive tuning parameter is the critical value of two-sample testing, which can be chosen by a BH-like procedure introduced in Wang (2023). See the detailed discussion in the Supplementary Material.

## 3 Results

### 3.1 Borrowing strength from neighborhood can increase power

In this section, we study the numerical performance of the MsRDB test through a series of experiments on simulated data. These numerical experiments aim to characterize how borrowing the neighborhood’s strength can boost the power of the differential abundance test. In these simulation studies, we simulate the ASV data from a dataset collected in Yatsunenko et al. (2012). With the simulated dataset, we compare the FDR and power of the MsRDB test with different versions of the RDB tests and several state-of-art differential abundance tests.

The first set of simulation studies aims to investigate ASV aggregation’s effect by comparing different versions of RDB tests. In particular, we consider the vanilla RDB test, the *k*-nearest neighbors weighted RDB test, and the MsRDB test. The results of FDR and power are summarized in Supplementary Figs S2, S7, and S8. These results suggest that the power is significantly improved if we incorporate information from the ASVs’ neighborhoods. In particular, the *k*-nearest neighbors weighted RDB test and the MsRDB test have larger power than the RDB test in all simulation experiments. When the number of neighbors increases, the power of both tests also increases since more information is borrowed from neighbors. However, simple incorporation from similar ASVs in the *k*-nearest neighbors weighted RDB test can lead to a highly inflated false discovery. Specifically, the FDR level can be as high as more than 50% sometimes. The inflation of false discovery is mainly because the nondifferentially abundant ASVs can be falsely identified when they aggregate information from some differentially abundant neighbors. Unlike the RDB test and *k*-nearest neighbors weighted RDB test, the MsRDB test can help improve power while controlling FDR at the desired level since the PS approach only allows sharing information between ASVs with similar levels of differential abundance. Note that the FDR in the MsRDB test may be slightly inflated when the number of neighbors becomes larger. In conclusion, aggregating information from the neighborhood can increase power, but controlling false discovery needs smart aggregation.

These simulation experiments in Supplementary Figs S2, S7, and S8 also help us investigate the influence of sample size, signal strength, and the number of differentially abundant ASVs on these three versions of the RDB tests. When the sample size increases, all these tests become more powerful and achieve similar FDR (Supplementary Fig. S2). Unlike the sample size, the number of differentially abundant ASVs has a relatively small influence on the performance of these tests (Supplementary Fig. S8). Similar to sample size, a large signal strength can lead to more powerful tests, but a small signal strength can slightly inflate the FDR in the MsRDB test (Supplementary Fig. S7). The intuition behind this phenomenon is that it is more difficult for the PS approach to distinguish ASVs with different levels of differential abundance when the signal strength is small. Hence, the nondifferentially abundant ASVs are more likely to aggregate information from differentially abundant ASVs.

In the second set of simulation studies, we compare the MsRDB test with five state-of-art differential abundance tests, including the RDB test (Wang 2023), ANCOM.BC (Lin and Peddada 2020a), DACOMP (Brill et al. 2022), ALDEx2 (Fernandes et al. 2013), and StructFDR (Xiao et al. 2017). The performance of these six methods is evaluated by FDR and power at the ASV level. All methods can control FDR at the ASV level very well, no matter the differential abundant ASVs belonging to different genera, classes, or local ASV clusters (Supplementary Figs S3, S9, and S10). Due to ASVs aggregation, MsRDB and StructFDR are significantly more powerful than the other four methods in all experiments, consistent with the observation in the previous set of simulation studies. Compared with StructFDR, MsRDB has larger power as embedding ASVs into a metric space allows more flexibility in ASVs aggregation than using a phylogenetic tree. In addition, the MsRDB test can still control false discovery and maintain adequate power when the simulated ASV data have the measurement error and unbalanced sequencing depth (Supplementary Figs S11 and S12).

The last set of simulation studies aims to compare the performance of differential abundance tests at a resolution of chosen taxonomy rank, including genus and family. We consider six differential abundant tests: MsRDB, RDB with ASV as analysis unit, RDB with a genus (family) as analysis unit, ANCOM.BC with ASV as analysis unit, ANCOM.BC with a genus (family) as analysis unit, and StructFDR. A taxon is defined as a differential abundant taxon if at least one ASV belonging to this taxon is differential abundant. Unlike the previous experiments, the performance of the differential abundance test is evaluated by FDR and power at the genus (family) level. Despite the analysis unit, all methods can control the FDR very well (Supplementary Figs S4 and S13). We can observe that using the genus (family) as analysis units can lead to a more powerful test than using ASV when the sample size is small. It is also interesting to note that StructFDR and MsRDB can detect more differential abundant microbes than the other methods because of their ASV aggregating strategy and multiscale technique. The three sets of simulation studies suggest that a proper aggregation strategy can improve the power of differential abundant tests.

### 3.2 MsRDB detects differentially abundant microbes associated with immigration

To elucidate the differences between ASV-wise analysis, taxon-wise analysis at different ranks, and the MsRDB test, we apply these microbiome data analysis strategies to a gut microbiota dataset from an immigration effect study (Vangay et al. 2018). In particular, we focus on the pairwise comparisons between Hmong female individuals who were living in Thailand (HmongThai, *n* = 96) or were born in the USA but whose parents were born in Southeast Asia (Hmong2nd, *n* = 54), and European American female individuals (Control, *n* = 36). Through pairwise comparisons, we can identify the microbes associated with immigration. To conduct comparisons, we apply four different analysis strategies: ASV-wise analysis, taxon-wise analysis at the genus level, taxon-wise analysis at the class level, and the MsRDB test. In ASV-wise and taxon-wise analysis, we consider three differential abundance tests: ANCOM.BC (Lin and Peddada 2020a), RDB test (Wang 2023), and ALDEx2 (Fernandes et al. 2013).

Overall, the differential abundance analysis results suggest that Hmong2nd is very similar to Control, while there are some differences between HmongThai and the other two groups. Regardless of the choices for differential abundance test, there are fewer discoveries if we regard each ASV as an analysis unit (Supplementary Figs S5, S16, and S17). The low detection power in ASV-wise analysis is mainly because low counts at each ASV are not informative enough for an effective comparison. The power is significantly improved after aggregation based on taxonomy, but we still need to choose an appropriate rank for analysis. A lower taxonomy rank brings finer resolution but also results in throwing out a fair amount of sequence. Specifically, at least 5% of valuable sequences in more than half samples are assigned to the NA genus and thus removed from the analysis if we choose genus as analysis unit (Supplementary Fig. S5D). Compared with ASV-wise analysis, the genus-wise analysis leads to more discoveries. On the other hand, if a higher taxonomy rank is used, we can keep more sequences but could lose resolution. More concretely, only a tiny proportion (<1% in most samples) of sequences is removed from the class-wise analysis (Supplementary Fig. S5D). However, the class-wise analysis only reports differentially abundant microbes at the class level and loses the resolution. In particular, we can observe that the analysis at different ranks contradicts each other sometimes in Supplementary Fig. S5. For example, genus-wise analysis suggests that some genera in class *Coriobacteriia*, *Bacilli*, *Desulfovibrionia*, *Negativicutes*, and *Gammaproteobacteria* are differentially abundant in HmongThai and Control comparison, but class-wise analysis makes an opposite conclusion.

The MsRDB test combines the advantages of both ASV-wise analysis and taxon-wise analysis. Like ASV analysis, the MsRDB test utilizes all sequences and detects the differentially abundant microbes at ASV resolution. Since strength is borrowed from the neighborhood, the MsRDB test is more powerful and has more discoveries than ASV-wise or taxon-wise analysis. Importantly, most discoveries by all different methods are recovered by the MsRDB test, whether they are detected at ASV, genus, or class level (Supplementary Figs S5, S16, and S17). This phenomenon is mainly because the weights in the MsRDB test are constructed iteratively so that the MsRDB test can capture multiscale signals.

**Figure 3.**
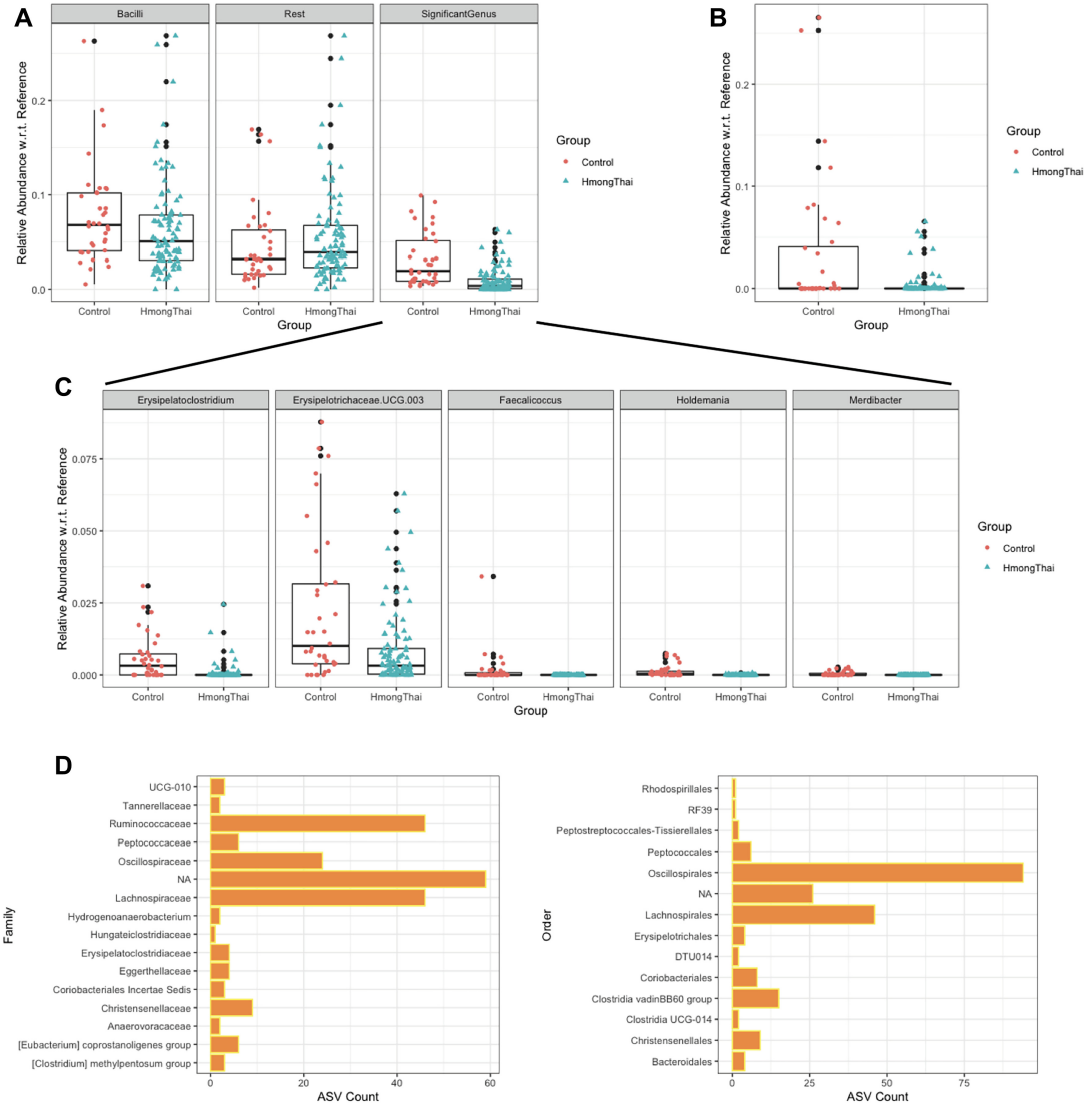
Taxa detected by different differential abundance analysis strategies on the real microbiota dataset. The reference set in all box plots is the collection of all nondifferentially abundant ASVs revealed by the MsRDB test. (A) The box plots show the relative abundance with respect to the reference set in class *Bacilli* (left), in five significant genera within *Bacilli* (right), and in the rest of genera within *Bacilli* (middle). (B) The box plot shows the relative abundance with respect to the reference set in significant ASVs within genus *Phascolarctobacterium*. (C) The relative abundance with respect to the reference set in five significant genera within *Bacilli* is shown. The five significant genera are identified by genus-wise analysis. (D) The family (left) and order (right) of significant ASVs assigned to the NA genus. These ASVs are identified by the MsRDB test and removed from the genus-wise analysis

The aggregation strategy in the taxon-wise analysis can help increase power but could also miss some heterogeneous signals. To illustrate this, we focus on the comparison between group HmongThai and Control. The class-wise analysis suggests that *Bacilli* is a nondifferentially abundant class (Fig. 3A). However, *Bacilli* is known to be associated with the western diet and is expected to be more abundant in the Control group (Clarke et al. 2012). Through the genus-wise analysis, we identify five differentially abundant genera in *Bacilli* (Fig. 3C). Figure 3A shows the box plot of the abundance in these five significant genera and the rest of the genera in *Bacilli*. These dominant nondifferentially abundant genera make it difficult to identify *Bacilli* in class-wise analysis. This example indicates we might miss some subtle heterogeneous signals in taxon-wise analysis.

Unlike taxon-wise analysis, the MsRDB test only aggregates ASVs with similar levels of differential abundance and thus can easily capture these heterogeneous signals. This special aggregation strategy partially explains why the MsRDB test identifies more genera or classes than taxon-wise analysis and potentially provides more biological insights into the microbial communities. In MsRDB analysis results, several ASVs assigned to genus *Phascolarctobacterium* are more abundant in group Control than HmongThai (Fig. 3B, present in 26% of HmongThai < present in 47% of Control), while it is nondifferentially abundant in genus-wise analysis. This discovery is consistent with previous results that *Phascolarctobacterium* is associated with a high-fat diet (Ariefdjohan et al. 2017). MsRDB analysis results suggest a decrease in the abundance of several ASVs assigned to *Ruminococcus torques* in HmongThai, compared with Control (Supplementary Fig. S15A, present in 21% of HmongThai < present in 78% of Control). A plant-based diet in Southeast Asia could explain the decrease in the abundance of *Ruminococcus torques* (Meslier et al. 2020). It is also interesting to observe that the rest of the ASVs assigned to *Ruminococcus torques* are not significantly differentially abundant, so genus-wise analysis finds it difficult to detect.

The ASVs removed from the taxon-wise analysis could also help to provide insight into the microbial communities. The MsRDB test identifies a few significant ASVs removed from genus-wise analysis in HmongThai and Control comparison. Supplementary Fig. S15B compares these significant ASVs’ abundance if we aggregate them together. There is a significant increase in Control compared with HmongThai. Although these significant ASVs are assigned to the NA genus, their taxonomy classification at family or order rank can still provide a rich source of information (Fig. 3D). Specifically, these genus-unassigned ASVs mainly come from the family *Ruminococcaceae*, *Oscillospiraceae*, and *Lachnospiraceae*, or order *Oscillospirales* and *Lachnospirales*.

### 3.3 MsRDB reveals microbial biogeography of wine grapes

To further demonstrate the performance of the MsRDB test, we apply it to a grape microbiota dataset (Bokulich et al. 2014). We consider studying the association between growing region and microbial community of grape Chardonnay. The grape must samples we consider here are divided into three groups based on their growing regions: 19 samples from Sonoma, 46 samples from Napa, and 11 samples from Central Coast. Unlike the previous dataset, a considerable amount of sequences in this dataset are assigned to the NA genus and family (Supplementary Fig. S6A). We need to remove these sequences from analysis if we adopt taxon-wise analysis to detect differentially abundant microbes at a resolution of genus or family. We apply the MsRDB test and ASV-wise analysis to explore differentially abundant microbes at an ASV resolution. Again, the MsRDB test is more powerful and can help detect more significant ASVs (Supplementary Fig. S18).

We cluster the differentially abundant ASVs into small groups since only partial information on the microbial taxonomy is known in this dataset. Supplementary Fig. S6B–D show the clustering results in pairwise comparisons between Sonoma, Napa, and Central Coast and indicates that the differentially abundant microbes naturally form around 10 small groups in each comparison. After comparing each small group with the assigned taxonomy, the small groups can correspond to different taxonomy ranks (Supplementary Table S1). For example, in comparing between Central Coast and Sonoma, ASVs in Cluster 1 come from the same genus *Lactobacillus*, while ASVs in Cluster 11 come from the same order *Burkholderiales*. This example again demonstrates that the MsRDB test can indeed capture multiscale signals. Although some ASVs lack assigned genus and family, they may still provide insight into microbial communities. For instance, ASVs Cluster 8 in the Sonoma and Napa comparison are assigned to NA genus and family, but the information in order *Enterobacterales* is still beneficial. The analysis in Bokulich et al. (2014) concludes that class *Actinobacteria* is more abundant in Central Coast than Napa. Our results confirm this finding and further suggest that the differentially abundant microbes come from family *Micrococcaceae* (Cluster 8), *Geodermatophilaceae* (Cluster 9), and *Kineosporiaceae* (Cluster 10) (Supplementary Fig. S6E).

## 4 Discussion

Differential abundance analysis is a challenging and complicated statistical problem because the observed dataset in microbiome studies only reflects the relative abundance of taxa, has excessive zeros, and is perturbed in experiments. Besides these major challenges, this work focuses on another important practical issue in differential abundance analysis: the choice of analysis unit. Through several numerical examples, we show that the analysis unit has a significant impact on the results of differential abundance analysis. On the one hand, the ASV-wise analysis can help detect differentially abundant microbes at the finest resolution but usually has small detection power. On the other hand, taxon-wise analysis enforces a tradeoff between resolution and sequence utility, although it can help increase detection power.

MsRDB test has both advantages of ASV-wise and taxon-wise analysis: it detects the differentially abundant microbes at a resolution of ASV, has larger detection power, and keeps all sequences in the analysis. Due to these good properties, the MsRDB test not only recovers most discoveries detected by conventional ASV-wise or taxon-wise analysis but also identifies some extra subtle differences between microbial communities. These new findings revealed by the MsRDB test could potentially provide more biological insights into the microbial communities and help identify new diagnostic or prognostic biomarkers in disease. As a generalization of the RDB test, findings reported by the MsRDB test is reliable and trustworthy as it is robust to compositional constraint, prevalent zero counts, and experimental bias in the microbial dataset. We hope this new differential abundance analysis method will help biological researchers in making more scientific discoveries.

This work not only develops a new differential abundance test but also suggests a change in how we utilize the ASVs’ sequence information in the differential abundance analysis. Conventional differential abundance analysis treats ASVs (or taxa) as independent units and thus does not take the most advantage of sequences’ information. In contrast, we show that embedding into a metric space makes it possible to exploit the spatial structure among ASVs’ sequences and significantly improve the power of differential abundance analysis. The MsRDB test presented in this work is mainly designed based on the RDB test because of its robustness. However, the perspective of embedding and spatial analysis is readily generalizable to other popular differential abundance analysis methods. For instance, after embedding into a metric space, the linear models used in ANCOM.BC can also work with the PS approach, as illustrated in Li et al. (2011). Therefore, we believe this new perspective will lead to more exciting methods for analyzing microbial compositional data.

The current MsRDB test mainly considers a standard two-sample *t*-test as test statistics and Gaussian distribution as its null distribution. As we illustrate in our numerical examples, such a strategy works very well when the sample size is large. However, when the sample size becomes small, the false discovery might not be controlled well. This problem might be alleviated by a rank-based test, such as the Wilcoxon test, as the null distribution of the rank-based test does not rely on the underlying distribution. Because of the two-sample *t*-test, the current MsRDB test is designed for testing the mean shift between groups. It would also be interesting to explore if the framework of the MsRDB test can be used to test other characteristics of microbial distribution, such as dispersion. Another potential extension of the MsRDB test is to study the association between microbial community and a continuous or multiple-level outcome, e.g. body mass index. This extension is possible if we consider replacing the *t*-test with a correlation test, as Wang (2023) illustrates.

## Supplementary Material

btad178_Supplementary_Data

## Data Availability

All three datasets can be downloaded from Qiita (https://qiita.ucsd.edu/). Dataset in Yatsunenko et al. (2012) is under study ID 850. Dataset in Vangay et al. (2018) is under study ID 12080. Dataset in Bokulich et al. (2014) is under study ID 2019.
