# Supplementary material for: Multiscale adaptive differential abundance analysis in microbial compositional data
Source: Bioinformatics. 2023 Apr 5;39(4):btad178. doi: 10.1093/bioinformatics/btad178 (PMC10112958; doi:10.1093/bioinformatics/btad178)
Supplement: btad178_Supplementary_Data [file btad178_supplementary_data.zip › MsRDB_Supp.pdf]

Supplementary Material to “Multi-scale Adaptive Differential Abundance Analysis in Microbial Compositional Data”

Shulei Wang*
University of Illinois at Urbana-Champaign

Abstract

In the Supplementary Material, we provide the detailed methods and extra results on numerical experiments.

S1 Methods Details

S1.1 Model for Microbiome Data

In this work, we consider the ASV counts data from $m_1 + m_2$ subjects, where m_1 subjects are in the first group, and m_2 are in the second group. Let $\mathcal{S}$ be the collection of observed distinct ASVs, and $s \in \mathcal{S}$ represents an ASV. For the jth subject in the kth group, we observe a vector of ASVs' counts $\mathbf{N}_{k,j} = \{\mathbf{N}_{k,j}(s) : s \in \mathcal{S}\}$, where $\mathbf{N}_{k,j}(s)$ is the number of ASV s observed in the subject, and a vector of covariates $\mathbf{X}_{k,j}$, such as age and gender. Since the total counts of ASVs observed from each subject do not reflect the absolute abundance, we usually normalize the count data from each subject as a vector of observed relative abundance $\hat{\mathbf{P}}_{k,j} = \{\hat{\mathbf{P}}_{k,j}(s) : s \in \mathcal{S}\}$, where $\hat{\mathbf{P}}_{k,j}(s) = \mathbf{N}_{k,j}(s)/\sum_{s\in\mathcal{S}} \mathbf{N}_{k,j}(s)$. In practice, such a vector of observed relative abundance is also called a compositional data vector. In differential abundance analysis, our goal is to compare the observed relative abundance from these two groups of subjects.

We assume the ASV counts data are drawn from the following model. For the jth subject in the kth group, there exists a latent vector of absolute abundance $\mathbf{A}_{k,j} = \{\mathbf{A}_{k,j}(s) : s \in \mathcal{S}\}$, which we do not observe in practice. We assume the absolute abundance vectors and covariates vectors are drawn from two populations

$$(\mathbf{A}_{k,1}, \mathbf{X}_{k,1}), \dots, (\mathbf{A}_{k,m_k}, \mathbf{X}_{k,m_k}) \sim \pi_k(\mathbf{A}, \mathbf{X}), \qquad k = 1, 2,$$

*Address for Correspondence: Department of Statistics, University of Illinois at Urbana-Champaign, 605 E. Springfield Ave., Champaign, IL 61820 (Email: shuleiw@illinois.edu).

where $\mathbf{X} \in \mathbb{R}^d$. $d = 0$ means that there are no observed covariates in the data set. These absolute abundance vectors $\mathbf{A}_{k,j}$ represent the abundance of ASVs in each subject. Our observed ASV counts data $\mathbf{N}_{k,j}$ are related to absolute abundance $\mathbf{A}_{k,j}$ through a relative abundance model with experimental bias. Specifically, we adopt the multiplicative factors model (McLaren et al., 2019) for experimental bias in relative abundance

$$\mathbf{P}_{k,j}(s) = \frac{\beta(s)\mathbf{A}_{k,j}(s)}{\sum_{s \in \mathcal{S}} \beta(s)\mathbf{A}_{k,j}(s)},$$

where $\beta(s)$ is the measurement efficiency factor for the ASV s, which could related to variation in multiple technical factors, such as rRNA extraction efficiency(Olson and Morrow, 2012) and PCR primer binding preference (Brooks et al., 2015). $\beta(s)$ is used to characterize the taxa specific bias and usually unknown in advance. Here, we want to emphasize that the relative abundance $\mathbf{P}_{k,j}(s)$ already takes the experimental bias into account. Given the relative abundance, we assume the ASV counts $\mathbf{N}_{k,j}$ is drawn from a multinomial model

$$\mathbf{N}_{k,j} | \mathbf{P}_{k,j}, N_{k,j} \sim \mathit{multinomial}(N_{k,j}, \mathbf{P}_{k,j}),$$

where $N_{k,j}$ is the total reads we observe for each subject. This model suggests that the observed relative abundance $\hat{\mathbf{P}}_{k,j}$ is just an empirical version of relative abundance $\mathbf{P}_{k,j}$.

S1.2 Reference-Based Hypothesis

We need to define a rigorous statistical hypothesis in differential abundance analysis. Although it seems that the definition of a hypothesis is straightforward, compositional constraints and experimental bias make it challenging to define a statistically identifiable and interpretable hypothesis. See more discussions in Wang (2023). The literature usually defines the statistical hypothesis based on a parametric model, but it might not be easy to interpret when the model is misspecified. This work defines a rigorous statistical hypothesis without assuming a parametric model. Specifically, we formulate the differential abundance analysis as a reference-based hypothesis testing problem (Brill et al., 2022).

We first introduce the concept of the reference set (Sohn et al., 2015; Brill et al., 2022). A subset of ASVs, $\mathcal{S}_0 \subset \mathcal{S}$, is called a reference set if $|\mathcal{S}_0| > |\mathcal{S}|/2$ and there exists a constant b such that

$$\mathbf{Q}_1(s) = b\mathbf{Q}_2(s), \qquad s \in \mathcal{S}_0, \tag{S1}$$

where $\mathbf{Q}_k(s) = \mathbb{E}(\mathbf{P}_{k,1}(s))$ is the expected relative abundance of ASV s in the kth group. Here $|\cdot|$ is the number of elements in the set. The definition of the reference set suggests that the expected relative abundance of ASVs in the reference set changes in the same amplitude across groups. In other words, the fold changes of ASVs' relative abundance in the reference set are the same. We define the reference set as (S1) because the relative abundance changes caused by compositional effect and experimental bias alone have the same amplitude. For example, in Figure S1, the relative abundance of orange, green, and blue components change across groups due to the compositional effect and experimental bias alone, but their relative abundances change in the same amplitude (37.5%/30%=18.75%/15%=6.25%/5%=1.25).

After introducing the reference set, we now define the reference-based hypothesis. The idea of the reference-based hypothesis is to consider the reference set as a benchmark and compare other ASVs with this benchmark. Specifically, we consider the following reference-based hypothesis at ASV s

$$H_{s,0}: \frac{\mathbf{Q}_1(s)}{\sum_{s\in\mathcal{S}_0}\mathbf{Q}_1(s)} = \frac{\mathbf{Q}_2(s)}{\sum_{s\in\mathcal{S}_0}\mathbf{Q}_2(s)} \qquad \text{v.s.} \qquad H_{s,1}: \frac{\mathbf{Q}_1(s)}{\sum_{s\in\mathcal{S}_0}\mathbf{Q}_1(s)} \neq \frac{\mathbf{Q}_2(s)}{\sum_{s\in\mathcal{S}_0}\mathbf{Q}_2(s)}. \tag{S2}$$

Since (S2) is defined for each ASV, testing the above hypothesis can identify the differential abundant microbes at ASV resolution. We can also write the hypothesis in (S2) as the following equivalent form

$$H_{s,0}: \mathbf{Q}_1(s) = b\mathbf{Q}_2(s) \qquad \text{v.s.} \qquad H_{s,1}: \mathbf{Q}_1(s) \neq b\mathbf{Q}_2(s),$$

where b is defined in the reference set. This above definition suggests that the reference-based hypothesis does not directly test whether the relative abundance changes across the two groups or not but aims to test if the fold change of each ASV is different from the majority of ASVs due to the assumption $|\mathcal{S}_0| > |\mathcal{S}|/2$. In the reference-based hypothesis, a change in the differential components can be interpreted as a change with respect to the collection of all non-differentiate ASVs. How does this reference-based hypothesis relate to the hypothesis defined by absolute abundance? If there exists a set of non-differential abundant ASVs (defined by the absolute abundance), this set of ASVs can be seen as a reference set. So testing the above reference-based hypothesis is roughly equivalent to directly testing if there is a change in absolute abundance.

A toy example is shown in Figure S1 to illustrate the idea of the reference set and reference-based hypothesis. In Figure S1, we have two ecosystems of red, orange, green, and blue components, A and B, and their absolute abundances are shown on the left-hand side. Due to the sampling process and experimental bias, the observed counts and relative abundance, shown on the right-hand side, cannot honestly reflect the relative relationship between different components' absolute abundance. However, despite the experimental bias, the reference-based hypothesis framework suggests that the orange, green, and blue components can be seen as a reference set since their relative abundances change in the same amplitude (37.5%/30%=18.75%/15%=6.25%/5%=1.25). Based on this reference set, only the red component belongs to the alternative hypothesis as its fold change differs from the other three (37.5%/50% $\neq$ 1.25), and the orange, green, and blue components belong to the null hypothesis. These conclusions are consistent with the fact that there is only a change in the red component's absolute abundance. This toy example shows that the reference-based hypothesis is a statistically identifiable and interpretable hypothesis for differential abundance analysis.

S1.3 Multiscale Adaptive Robust Differential Abundance Analysis (MsRDB)

To test the reference-based hypothesis in (S2), we introduce a multi-scale adaptive robust differential abundance test (MsRDB). In the MsRDB test, the input is the ASV counts table

and the corresponding ASVs' sequences. Given the input, the roadmap of the MsRDB test consists of the following three parts:

1. Initialization: the ASV counts table is transformed into the relative abundance table. The ASVs' sequences are used to evaluate the ASV distance matrix. The details of the ASV distance matrix are explained in Section S1.3.1.

2. Weights calculation: evaluate weights $w(s; s')$ by applying propagation and separation approach to ASV relative abundance table and ASV distance matrix. The output of this step is a weighted ASV table. The details are explicated in Section S1.3.2.

3. Differential abundant ASV detection: identify differential abundant ASVs by applying the weighted RDB test to the weighted ASV relative abundance table. The details are explicated in Section S1.3.3.

Through these three parts, the MsRDB test identifies a collection of differentially abundant ASVs. Note that the analysis of the MsRDB test does not need information taxonomy, while the interpretation results may still need it. For the choices of tuning parameters in this algorithm, see Section S1.4.3.

S1.3.1 Embedding of ASVs' Sequences

Different ASVs are not equally distinct, as some are more similar than others. Motivated by this observation, we can consider a general way to incorporate the similarity between ASVs. Specifically, we opt to embed different ASVs into a metric space and define a distance between ASVs' raw sequences $d(\cdot, \cdot)$. To reflect different aspects of ASVs, we provide several different possible ways to calculate the distance between two distinct ASV sequences:

- *Pairwise alignment distance.* One commonly used way to compare two sequences is pairwise sequence alignment, e.g., the Needleman-Wunsch algorithm. After alignment, the scoring schemes in the alignment algorithm can be used to calculate the distance between sequences.

- *$k$mer-based distance.* Another way to compare two sequences is $k$mer-based distance. Given a positive integer k, each sequence is transformed to a vector of $k$mer counts. Then, a distance between two $k$mer count vectors is used to measure the dissimilarity of sequences. For example, Euclidian squared, and Mahalanobis distances are two of the most commonly used distances (Gentleman and Mullin, 1989).

- *Phylogenetic distance.* If the evolutionary history of sequences is believed to relate to the outcome of interest, we can also use the existing phylogenetic tree to measure the dissimilarity of sequences. Specifically, the sequences are placed on a reference phylogenetic tree by phylogenetic placement algorithms, such as SEPP (Janssen et al., 2018). The distance between the sequences can then be defined as the length of the unique path connecting them on the tree.

This list of distance options could be incomplete, as some other information can also be incorporated into the distance. For example, the sequence can also be used to predict gene families or pathways (Douglas et al., 2020), and they can define the distance between sequences. In addition, a weighted sum of several distances could also be used when needed. In most of the analyses in this work, the distances between ASVs are calculated by function `DistanceMatrix` in `DECIPHER` R package (Wright, 2016). The output of this step is a distance matrix $\{d(s, s')\}_{s,s' \in \mathcal{S}}$.

S1.3.2 Multiscale Adaptive Weights Construction

In taxon-wise analysis, ASVs are aggregated by assigned taxonomy since the ASVs assigned to the same taxon are believed to be similar. Instead of using taxonomy, we assign weights $w(s, s')$ to each pair of ASVs to capture their similarity. Compared with taxonomy-based aggregation, the weights provide more flexibility in ASV aggregation and avoid throwing out the unassigned sequence in taxonomic classification. The general goal of weights construction is to increase detection power by borrowing strength from the neighborhood and reduce false discoveries by avoiding aggregation of differential and non-differential abundant ASVs.

Form of weights. One important advantage of weights $w(s, s')$ is that they can incorporate different types of similarity information between ASVs. More specifically, the weights we use here reflect two types of information: how different the two ASVs' sequences are and how different the levels of differential abundance are. Compared with the classical form of weights in nonparametric statistics, our weights also reflect the similarity of differential abundance levels. The rationale for incorporating differential abundance levels is to ensure ASVs aggregation with similar levels of differential abundance. Specifically, the form of weights we consider is

$$w(s; s') = \mathbf{K}_s\left(\frac{d(s, s')}{r}\right)\mathbf{K}_a\left(\frac{D(\widehat{DA}(s); \widehat{DA}(s'))}{h}\right). \tag{S3}$$

Here, $\mathbf{K}_s$ and $\mathbf{K}_a$ are two non-negative and non-increasing kernels with compact support, e.g., $\mathbf{K}_a(x) = \max(1 - x, 0)$ and $\mathbf{K}_s(x) = \mathbf{I}(x < 1)$, where $\mathbf{I}$ is an indicator function. $\mathbf{K}_s$ and $\mathbf{K}_a$ are used to incorporate different types of information:

- $\mathbf{K}_s$ is a kernel used to measure sequence similarity. In the kernel $\mathbf{K}_s$, $d(s, s')$ is the distance between ASVs' sequences introduced in Section S1.3.1, and r is a tuning parameter to control the size of the neighborhood. We only aggregate the information from the ASVs which share similar sequences through the kernel $\mathbf{K}_s$.

- $\mathbf{K}_a$ is a kernel used to measure differential abundance level similarity. The reference-based hypothesis suggests that the level of differential abundance can be captured by fold change across the groups. We introduce a robust coefficient of fold change to measure the level of differential abundance

$$DA(s) = \frac{\mathbf{Q}_1(s) - \mathbf{Q}_2(s)}{\mathbf{Q}_1(s) + \mathbf{Q}_2(s)}$$

where $\mathbf{Q}_k(s) = \mathbb{E}(\mathbf{P}_{k,1}(s))$ is the expected relative abundance of ASV s in the kth group. All non-differential abundant ASVs have $DA(s) = (b-1)/(b+1)$ and thus share the same level of differential abundance, where b is the constant in the definition of the reference set. Since $DA(s)$ cannot be measured directly, we introduce the corresponding estimator of $DA(s)$

$$\widehat{DA}(s) = \frac{\bar{\mathbf{P}}_{1,w}(s) - \bar{\mathbf{P}}_{2,w}(s)}{\bar{\mathbf{P}}_{1,w}(s) + \bar{\mathbf{P}}_{2,w}(s)},$$

where $\bar{\mathbf{P}}_{k,w}(s)$ is the weighted mean of the relative abundance of ASV s in the kth group, i.e., $\bar{\mathbf{P}}_{k,w}(s) = \sum_{s' \in \mathcal{S}} w(s;s')\bar{\mathbf{P}}_k(s')$. Here $\bar{\mathbf{P}}_k(s)$ is the mean of the relative abundance of ASV s in the kth group. To compare the differential abundance level, we define the following distance between s and s'

$$D(\widehat{DA}(s); \widehat{DA}(s')) = \frac{|\widehat{DA}(s) - \widehat{DA}(s')|}{[\hat{\sigma}^2_{DA}(s)]^{1/2}},$$

where $\hat{\sigma}^2_{DA}(s)$ is an estimator for the variance of $\widehat{DA}(s)$. h in $\mathbf{K}_a$ is a tuning parameter to penalize the similarity of differential abundance between ASVs.

Propagation and separation. There are two challenges to evaluating the weights in (S3): 1) the definition of $w(s;s')$ and $\widehat{DA}(s)$ rely on each other, so we need to evaluate them simultaneously; 2) the performance of the weighted method usually depends on the choice of neighborhood size r and the optimal choice of r could be different at different ASVs. To alleviate these challenges, we adopt an iterative and multiscale approach, propagation and separation method (PS) (Polzehl and Spokoiny, 2000, 2006), to select neighborhood size and update estimation for $w(s;s')$ and $\widehat{DA}(s)$ simultaneously. The PS approach has been widely used in various imaging analyses (Li et al., 2011; Wang et al., 2019).

In the PS approach, we first choose a sequence of increasing radii, $0 = r_0 < r_1 < \ldots < r_v < \ldots < r_V$. We let radius $r_0 = 0$ and weights $w(s;s';0) = \mathbf{I}(s = s')$ initialize the process, where $\mathbf{I}(\cdot)$ is an indicator function. At the vth iteration, we first update the estimator for the level of differential abundance by the weight $w(s;s';v-1)$

$$\widehat{DA}(s;v) = \frac{\bar{\mathbf{P}}_{1,w}(s;v) - \bar{\mathbf{P}}_{2,w}(s;v)}{\bar{\mathbf{P}}_{1,w}(s;v) + \bar{\mathbf{P}}_{2,w}(s;v)},$$

where the weighted mean of relative abundance is estimated by $w(s;s';v-1)$

$$\bar{\mathbf{P}}_{k,w}(s;v) = \sum_{s' \in \mathcal{S}} w(s;s';v-1)\bar{\mathbf{P}}_k(s').$$

After updating $\widehat{DA}(s;v)$, we can then update the weights

$$w(s;s';v) = \mathbf{K}_s\left(\frac{d(s,s')}{r_v}\right)\mathbf{K}_a\left(\frac{D(\widehat{DA}(s;v);\widehat{DA}(s';v))}{h}\right).$$

We can update the weights and differential abundance level alternatively.

When does this iterative procedure stop? The stopping criteria for the above iterative procedure are based on the estimated level of differential abundance. Specifically, we choose the V_L as the number of required iterations and V_U as the largest possible iteration number. We shall not stop the PS approach when $v \leq V_L$ and always end it when $v = V_U$. When $V_L < v \leq V_U$, we compare $\widehat{DA}(s; v)$ with $\widehat{DA}(s; V_L)$ and stop if

$$D\left(\widehat{DA}(s; v); \widehat{DA}(s; V_L)\right) < H.$$

After the PS approach stops, we collect the weights in the final iteration and use them to construct a weighted abundance table.

S1.3.3 Weighted RDB test

After ASV aggregation by the weights in Section S1.3.2, we can test the reference-based hypothesis in (S2) on the weighted abundance table. To be robust to the zero counts, we adopt the iterative empirical Bayes framework introduced in Wang (2023). We first review the idea of the iterative empirical Bayes framework and then introduce the extension of this idea on the weighted abundance table.

Vanilla RDB test with infinite samples. To introduce the iterative empirical Bayes framework, we consider an ideal setting where the number of observed samples is infinite. In other words, we assume that $\mathbf{Q}_1(s)$ and $\mathbf{Q}_2(s)$ are observed in advance. The key observation in the iterative empirical Bayes framework is that the fold changes of non-differential abundant ASVs are always the same regardless of renormalization. Putting it mathematically, for any subset $\mathcal{S}'$ such that $\mathcal{S}' \subset \mathcal{S}$, there exists a constant b' such that

$$\frac{\mathbf{Q}_1(s)}{\sum_{s' \in \mathcal{S}'} \mathbf{Q}_1(s')} = b' \frac{\mathbf{Q}_2(s)}{\sum_{s' \in \mathcal{S}'} \mathbf{Q}_2(s')}, \qquad s \in \mathcal{S}_0. \tag{S4}$$

Motivated by this observation, we introduce the vanilla RDB test with infinite samples. In the RDB test, we define the active set $\mathcal{S}_{(t)}$ and differential abundant set $\mathcal{S}^*_{(t)}$ at the tth iteration. To initialize the process, we choose $\mathcal{S}^*_{(0)} = \emptyset$ and $\mathcal{S}_{(0)} = \mathcal{S}$. In the tth iteration, we renormalize the relative abundance with respect to the active set $\mathcal{S}_{(t-1)}$

$$\mathbf{Q}_1(s|\mathcal{S}_{(t-1)}) = \frac{\mathbf{Q}_1(s)}{\sum_{s' \in \mathcal{S}_{(t-1)}} \mathbf{Q}_1(s')} \qquad \text{and} \qquad \mathbf{Q}_2(s|\mathcal{S}_{(t-1)}) = \frac{\mathbf{Q}_2(s)}{\sum_{s' \in \mathcal{S}_{(t-1)}} \mathbf{Q}_2(s')}.$$

The observation in (S4) suggests that $\mathbf{R}(s|\mathcal{S}_{(t-1)}) = \mathbf{Q}_1(s|\mathcal{S}_{(t-1)}) - \mathbf{Q}_2(s|\mathcal{S}_{(t-1)})$ has the same sign for non-differential abundant ASVs. Since $|\mathcal{S}_0| > |\mathcal{S}|/2$ in the definition of the reference set, we can conclude

$$\text{sign}(\mathbf{R}(s|\mathcal{S}_{(t-1)})) = \text{sign}(\text{Median}\{\mathbf{R}(s|\mathcal{S}_{(t-1)}), s \in \mathcal{S}_{(t-1)}\}), \qquad s \in \mathcal{S}_0.$$

Equivalently, if we define

$$\tilde{\mathcal{S}}_{(t)} = \left\{s \in \mathcal{S}_{(t-1)} : \text{sign}(\mathbf{R}(s|\mathcal{S}_{(t-1)})) \neq \text{sign}(\text{Median}\{\mathbf{R}(s|\mathcal{S}_{(t-1)}), s \in \mathcal{S}_{(t-1)}\})\right\},$$

we can conclude ASVs in $\tilde{\mathcal{S}}_{(t)}$ are differential abundant ASVs. Then we can update the active set and differential abundant set

$$\mathcal{S}_{(t)} = \mathcal{S}_{(t-1)} \setminus \tilde{\mathcal{S}}_{(t)} \qquad \text{and} \qquad \mathcal{S}^*_{(t)} = \mathcal{S}^*_{(t-1)} \cup \tilde{\mathcal{S}}_{(t)}.$$

We can repeat this procedure and stop when $\tilde{\mathcal{S}}_{(t)} = \emptyset$. If the last iteration is T, the estimator for $\mathcal{S}_0 = \{s : \mathbf{Q}_1(s) = b\mathbf{Q}_2(s)\}$ and $\mathcal{S}_1 = \{s : \mathbf{Q}_1(s) \neq b\mathbf{Q}_2(s)\}$ are $\hat{\mathcal{S}}_0 = \mathcal{S}_{(T)}$ and $\hat{\mathcal{S}}_1 = \mathcal{S}^*_{(T)}$. When we observe infinite samples, Wang (2023) proves that the RDB test can identify all differential abundant ASVs accurately, i.e., $\hat{\mathcal{S}}_0 = \mathcal{S}_0$ and $\hat{\mathcal{S}}_1 = \mathcal{S}_1$.

Vanilla RDB test with finite samples. In practice, we only observe finite samples and need to redefine the RDB test to account for the randomness in data. Specifically, we can replace the sign comparison with the directional two-sample testing in $\tilde{\mathcal{S}}_{(t)}$. To see the connection between the sign comparison and the directional two-sample testing, we rewrite $\tilde{\mathcal{S}}_{(t)}$ in the following way

$$\tilde{\mathcal{S}}_{(t)} = \begin{cases} \left\{s \in \mathcal{S}_{(t-1)} : \mathbf{R}(s|\mathcal{S}_{(t-1)}) \leq 0\right\}, & M(\mathcal{S}_{(t-1)}) > 0 \\ \left\{s \in \mathcal{S}_{(t-1)} : \mathbf{R}(s|\mathcal{S}_{(t-1)}) \neq 0\right\}, & M(\mathcal{S}_{(t-1)}) = 0 \\ \left\{s \in \mathcal{S}_{(t-1)} : \mathbf{R}(s|\mathcal{S}_{(t-1)}) \geq 0\right\}, & M(\mathcal{S}_{(t-1)}) < 0 \end{cases},$$

where $M(\mathcal{S}_{(t-1)}) = \text{Median}\{\mathbf{R}(s|\mathcal{S}_{(t-1)}), s \in \mathcal{S}_{(t-1)}\}$. The above form of $\tilde{\mathcal{S}}_{(t)}$ suggests the empirical Bayes interpretation of this method: 1) we first look at the distribution of $\mathbf{R}(s|\mathcal{S}_{(t-1)})$ to infer testing direction; 2) we conduct the directional two-sample test at each s. Due to the finite samples, we can evaluate t-test statistics on the relative abundance instead of $\mathbf{R}(s|\mathcal{S}_{(t)})$

$$\hat{\mathbf{R}}(s|\mathcal{S}_{(t)}) = \frac{\bar{\mathbf{P}}_1(s|\mathcal{S}_{(t)}) - \bar{\mathbf{P}}_2(s|\mathcal{S}_{(t)})}{[\hat{\sigma}_1^2(s|\mathcal{S}_{(t)})/m_1 + \hat{\sigma}_2^2(s|\mathcal{S}_{(t)})/m_2]^{1/2}},$$

where $\bar{\mathbf{P}}_k(s|\mathcal{S}_{(t)})$ and $\hat{\sigma}_k^2(s|\mathcal{S}_{(t)})$ are the mean and variance of renormalized relative abundance. By replacing it with t-test statistics, we can redefine $\tilde{\mathcal{S}}_{(t)}$ as the result of directional two-sample testing

$$\tilde{\mathcal{S}}_{(t)} = \begin{cases} \left\{s \in \mathcal{S}_{(t-1)} : \hat{\mathbf{R}}(s|\mathcal{S}_{(t-1)}) \leq -D\right\}, & \hat{M}(\mathcal{S}_{(t-1)}) > M \\ \left\{s \in \mathcal{S}_{(t-1)} : |\hat{\mathbf{R}}(s|\mathcal{S}_{(t-1)})| > D\right\}, & -M \leq \hat{M}(\mathcal{S}_{(t-1)}) \leq M \\ \left\{s \in \mathcal{S}_{(t-1)} : \hat{\mathbf{R}}(s|\mathcal{S}_{(t-1)}) > D\right\}, & \hat{M}(\mathcal{S}_{(t-1)}) < -M \end{cases},$$

where $\hat{M}(\mathcal{S}_{(t-1)}) = \text{Median}\{\hat{\mathbf{R}}(s|\mathcal{S}_{(t-1)}), s \in \mathcal{S}_{(t-1)}\}$. Here $M > 0$ is a threshold for the median, and $D > 0$ is the critical value for the directional two-sample test. Due to the iterative nature, controlling false discovery in the RDB test is slightly different from conventional multiple testing settings (p-values are not updated iteratively in conventional settings). We need to choose M and D to control the false discoveries, and their choices are discussed in Wang (2023). It is worth noting that the design of the RDB test allows some correlation between

test statistics since independence structure is not a reasonable assumption for microbiome data due to the negative correlation in the compositional data and some strong dependence between microbial species. Compared with classical differential abundance analysis, the RDB test can simultaneously handle the zero counts and compositionality of data.

Weighted RDB test. Since we already assign the weights between ASVs, we consider utilizing information from similar ASVs to determine the sign of $\mathbf{R}(s|\mathcal{S}_{(t)})$. Instead of using the standard two-sample t-test, we adopt weighted t-test statistics

$$\hat{\mathbf{R}}_w(s|\mathcal{S}_{(t)}) = \frac{\bar{\mathbf{P}}_{1,w}(s|\mathcal{S}_{(t)}) - \bar{\mathbf{P}}_{2,w}(s|\mathcal{S}_{(t)})}{[\hat{\sigma}^2_{1,w}(s|\mathcal{S}_{(t)})/m_1 + \hat{\sigma}^2_{2,w}(s|\mathcal{S}_{(t)})/m_2]^{1/2}}.$$

Here, $\bar{\mathbf{P}}_{k,w}(s|\mathcal{S}_{(t)})$ and $\hat{\sigma}^2_{k,w}(s|\mathcal{S}_{(t)})$ are weighted mean and variance of weighted renormalized relative abundance at ASV s, defined as

$$\hat{\mathbf{P}}_{k,j,w}(s|\mathcal{S}_{(t)}) = \sum_{s' \in \mathcal{S}} w(s;s') \frac{\hat{\mathbf{P}}_{k,j}(s')}{\sum_{s'' \in \mathcal{S}_{(t)}} \bar{\mathbf{P}}_k(s'')}.$$

Through borrowing strength from its neighborhood, weighted t-test statistics are more powerful than standard t-test statistics. Given the weighted t-test statistics, we can replace all standard t-test statistics with weighted t-test statistics in $\tilde{\mathcal{S}}_{(t)}$

$$\tilde{\mathcal{S}}_{(t)} = \begin{cases} \left\{ s \in \mathcal{S}_{(t-1)} : \hat{\mathbf{R}}_w(s|\mathcal{S}_{(t-1)}) \leq -D \right\}, & \hat{M}_w(\mathcal{S}_{(t-1)}) > M \\ \left\{ s \in \mathcal{S}_{(t-1)} : |\hat{\mathbf{R}}_w(s|\mathcal{S}_{(t-1)})| > D \right\}, & -M \leq \hat{M}_w(\mathcal{S}_{(t-1)}) \leq M \\ \left\{ s \in \mathcal{S}_{(t-1)} : \hat{\mathbf{R}}_w(s|\mathcal{S}_{(t-1)}) > D \right\}, & \hat{M}_w(\mathcal{S}_{(t-1)}) < -M \end{cases},$$

where $\hat{M}_w(\mathcal{S}_{(t-1)}) = \text{Median}\{\hat{\mathbf{R}}_w(s|\mathcal{S}_{(t-1)}), s \in \mathcal{S}_{(t-1)}\}$. After using weighted t-test statistics, we have a new method called the weighted RDB test. In the weighted RDB test, we can still apply the same false discovery control mechanism as the original RDB test since its design allows some correlation between test statistics.

S1.4 Remarks on MsRDB Test

S1.4.1 Results Interpretation in MsRDB Test

To transfer the results obtained by the MsRDB test into knowledge, we still need to interpret these differentially abundant ASVs. We consider two aspects of MsRDB results interpretation: abundance change and taxonomy assignment.

- (**Abundance change**) The framework of the reference-based hypothesis provides a natural way to interpret the results obtained from the compositional data. Specifically, we can regard the collection of non-differential ASVs $\hat{\mathcal{S}}_0$ as a reference set and compare

everything with this estimated reference set. More specifically, we can always compare the relative abundance with respect to the reference set directly

$$\mathbf{P}_{k,j}(s|\hat{\mathcal{S}}_0) = \frac{\mathbf{P}_{k,j}(s)}{\sum_{s' \in \hat{\mathcal{S}}_0} \mathbf{P}_{k,j}(s')}, \qquad j = 1, \ldots, m_k \text{ and } k = 1, 2.$$

A change in the compositional data is interpreted as a change with respect to the estimated reference set. In the box plots of this work, we always plot the relative abundance with respect to the reference $\mathbf{P}_{k,j}(s|\hat{\mathcal{S}}_0)$ rather than the original relative abundance $\mathbf{P}_{k,j}(s)$.

- (**Taxonomy assignment**) Since we may find difficulty in assigning taxonomy to some of the ASVs, we opt first to cluster these differentially abundant ASVs into small groups. Specifically, we construct a graph for differentially abundant ASVs by thresholding the ASV distance matrix and then apply a graph-based clustering method for the resulting graph. After the small groups are constructed, we assign taxonomy to each small group of differential ASVs. The benefit of assigning taxonomy to the clusters is that it allows detecting unknown differentially abundant ASVs and assigns different taxonomy rank to different ASV groups.

S1.4.2 Covariate Balancing

In an observational study, we also observe several additional covariates $\mathbf{X}_{k,j}$, which might be related to treatment assignment and compositional data. the MsRDB test can work with covariate balancing techniques, such as the weighting method (Imbens and Rubin, 2015), to reduce the potential confounding effect. The weighting method here is different from the ASV-weighted method discussed in the previous section. The weighting method aims to assign weights for each subject so that the distributions of the covariates in each population are roughly the same. Specifically, after the weights $w_{k,j}$ are assigned to each subject, we can consider a subject-weighted version of mean and variance instead of standard mean and variance in the original MsRDB test. In particular, we choose the empirical balancing calibration weighting method (CAL) proposed by Chan et al. (2016), which provides a nonparametric way to estimate weights.

S1.4.3 Practical Considerations

To implement the MsRDB test, we need to specify the choices of several tuning parameters.

- **(multiscale adaptive weights)** We need to choose the kernel $\mathbf{K}_s$ and radii $r_1 < \ldots < r_V$ together. To simplify the implementation, we adopt the idea from the k-nearest neighbor algorithm. Specifically, we choose $K_s(x) = \mathbf{I}(x < 1)$. The radius r_v at ASV s is chosen in the following way: we first sort the distance $d(s, s')$ in a sense that

$$d(s, s'_{(1)}) \leq d(s, s'_{(2)}) \leq \ldots \leq d(s, s'_{(|\mathcal{S}|)});$$

the radius $r_v = d(s, s'_{(k_v)})$ is chosen for a predefined integer k_v. Equivalently, we include k_v neighbors at the iteration v and assign the weights of these k_v neighbors as 1. We choose k_v as a geometric sequence, that is, $k_v = \lceil k^{v/V} \rceil$, where $V = V_U$ is the largest number of iterations, and k is the number of neighbors in the last iteration. The main advantage of using a strategy in the k-nearest neighbor algorithm is that the total number of weights we need to store is just $|\mathcal{S}| \times k$, so the computation complexity of the weights update is also $|\mathcal{S}| \times k$.

There are many different options for $\mathbf{K}_a$ and h. Based on our experience, we choose $\mathbf{K}_a(x) = \exp(-2x^2)$ and $h = 10\sqrt{\log(|\mathcal{S}|)}$ in our implementation. These parameters are mainly used to separate the ASVs with different levels of differential abundance. In the stopping criteria, we choose $V_L = 5$, $H = 2\sqrt{\log(|\mathcal{S}|)}$ and $V_U = V = 10$. V_L cannot be set as a too small number, e.g. 1, since the first a few iterations might not be stable. The computational complexity relies on V_U, as it controls the number of iterations we need to evaluate. See more discussions in Li et al. (2011); Wang et al. (2019).

- **(weighted RDB test)** In the weighted RDB test, we use the same choices of parameters as the original RDB test. As we always aim to control FDR, the critical value for directional testing is chosen adaptively, as discussed in Section 4 of Wang (2023). The covariate balancing method we used in the MsRDB test is the weighting method implemented in `ATE` R package.

S1.5 Numerical Experiment Setup

S1.5.1 Simulation Setup

The simulated ASV data set is generated from a real gut microbiota data set collected in Yatsunenko et al. (2012). This data set includes V4 16S rRNA data from 528 subjects. The data set is trimmed at length 100 and denoised by Deblur method (Amir et al., 2017).

Simulation Study I We keep 1965 ASVs which appear in more than 3% of all subjects. To simulate the data set, we consider the following procedure:

1. We randomly draw m subjects as the treated group and m subjects as the control group.

2. We randomly select s ASVs and their neighbors as differential ASVs. The distance between ASVs is defined by the hamming distance between aligned sequences and calculated by function `DistanceMatrix` in `DECIPHER` R package. The number of neighbors is selected randomly between 10 and 15.

3. For each selected differential ASV and its neighbors, the signal strength is randomly chosen between $1 + \lambda$ and $1 + 2\lambda$. The count data in the treated group are multiplied by the chosen signal strength.

4. To mimic the experimental bias, we multiply the count data of each ASV by a measurement efficiency constant randomly drawn between 1 and 10.

In this simulation setup, m is the sample size, λ is the signal strength, and s is the number of differential ASVs (sparsity). Specifically, we consider three sets of simulation experiments:

- We first investigate the influence of sample size, so we choose $m = 50, 100, 200, 400$, $\lambda = 10$, and $s = 20$. The results are summarized in Figure S2.
- We next investigate the influence of signal strength, so we choose $m = 300$, $\lambda = 2, 4, 6, 8$, and $s = 20$. The results are summarized in Figure S7.
- Finally, we investigate the influence of the number of differential ASVs, so we choose $m = 200$, $\lambda = 8$, and $s = 5, 10, 15, 20$. The results are summarized in Figure S8.

Simulation Study II We keep 859 ASVs which appear in more than 10% of all subjects. To simulate the data, we adopt the same procedure as simulation study I with the following modification:

- In Figure S3, the differential ASVs are all ASVs in genera *Ruminococcus*, *Bacteroides*, *Faecalibacterium*, *Oscillibacter*, and *Blautia*. The signal strength of each genus is randomly chosen between $1 + \lambda$ and $1 + 2\lambda$ with $\lambda = 20$. There is no measurement error.
- In Figure S9, the differential ASVs are all ASVs in classes *Bacteroidia* and *Bacilli*. The signal strength of each class is randomly chosen between $1+\lambda$ and $1+2\lambda$ with $\lambda = 20$. There is no measurement error.
- In Figure S10, the differential ASVs are $s = 5$ ASV clusters chosen as Step 2. The number of neighbors is selected randomly between 5 and 10. The signal strength of each class is randomly chosen between $1 + \lambda$ and $1 + 2\lambda$ with $\lambda = 20$. There is no measurement error.
- In Figure S11, the setting is the same with Figure S10, but there is measurement error as Step 4.
- In Figure S12, the setting is the same with Figure S11, but the sequencing depth is multiply by η in the first group, where η is randomly chosen as 2 or 3.

In this set of simulation experiment, we consider the following methods and tuning parameters

- MsRDB: $k = 10$ and other tuning parameters are default values.
- RDB: all tuning parameters are default values.
- ANCOM.BC: multiple testing method is "holm" (same with its tutorial) and the pseudo-count is 1. Other tuning parameters are default values.

- DACOMP: the pseudo-count is 1 in reference selection, the test is `DACOMP.TEST.NAME.WILCOXON`, and multiple testing method is “BH”. Other tuning parameters are default values.

- ALDEx2: multiple testing method is “BH” and other tuning parameters are default values.

- StructFDR: the phylogenetic tree is estimated by UPGMA method (implemented by `upgma` in `phangorn` R package) and the distance matrix is calculated by function `DistanceMatrix` in `DECIPHER` R package. Set raw.count as TRUE and other tuning parameters are default values.

Simulation Study III In this set of simulation experiment, we follow the same procedure in Figure S10 with $s = 10$ ASV clusters as differential abundant ASVs. The true differential abundant genus (family) is a genus (family) with at least one differential abundant ASV.

We consider the following methods and tuning parameters

- MsRDB: $k = 10$ and other tuning parameters are default values. The method is applied to ASV table directly. All genera (families) including differential abundant ASVs are differential abundant genera (families).

- RDB-ASV: all tuning parameters are default values. The method is applied to ASV table directly. All genera (families) including differential abundant ASVs are differential abundant genera (families).

- RDB-Taxa: all tuning parameters are default values. The ASV table is converted into a genus (family) table and then the method is applied to genus (family) table.

- ANCOMBC-ASV: multiple testing method is “holm” (same with its tutorial) and the pseudo-count is 1. Other tuning parameters are default values. The method is applied to ASV table directly. All genera (families) including differential abundant ASVs are differential abundant genera (families).

- ANCOMBC-Taxa: multiple testing method is “holm” (same with its tutorial) and the pseudo-count is 1. Other tuning parameters are default values. The ASV table is converted into a genus (family) table and then the method is applied to genus (family) table.

- StructFDR: the phylogenetic tree is estimated by UPGMA method (implemented by `upgma` in `phangorn` R package) and the distance matrix is calculated by function `DistanceMatrix` in `DECIPHER` R package. Set raw.count as TRUE and other tuning parameters are default values. The method is applied to ASV table directly. All genera (families) including differential abundant ASVs are differential abundant genera (families).

S1.5.2 Differential Abundance Analysis in the Study of Immigration

Taxonomy in the immigration study data set (Vangay et al., 2018) is assigned by function `assignTaxonomy` in `dada2` R package with training files from the Silva Project's version 138.1 release. The differential abundance analysis includes 14471 ASVs assigned to kingdom *Bacteria*. Besides the microbiome data, we also observe several extra variables in this observational study. In particular, we include age and BMI into our analysis, as they might bring potential confounding effects. The degree of covariance balancing in age and BMI suggests that neither variable is well balanced, so we must adjust these two covariates in differential abundance analysis (Figure S14). We adopt the weighting method implemented in `ATE` R package for the RDB and the MsRDB tests to balance covariates. In the MsRDB test, the distance matrix between ASVs' sequences is calculated by function `DistanceMatrix` in `DECIPHER` R package. In the MsRDB test, the number of neighbors in the last iteration is $k = 20$.

S1.5.3 Differential Abundance Analysis in the Study of Wine Grape

Similar to the previous data set, we assign taxonomy of ASVs in wine grape data set (Bokulich et al., 2014) by function `assignTaxonomy` in `dada2` R package. The distance matrix in the MsRDB test is also calculated by function `DistanceMatrix` in `DECIPHER` R package. In the MsRDB test, the number of neighbors in the last iteration is $k = 15$. In clustering analysis, we first construct a graph where two significant ASVs are connected if they are in each other k nearest neighbor. Then, each connected subgraph is a cluster, and the small clusters with a total number of reads smaller than 10 are removed from the results.

S2 Extra Numerical Results and Figures

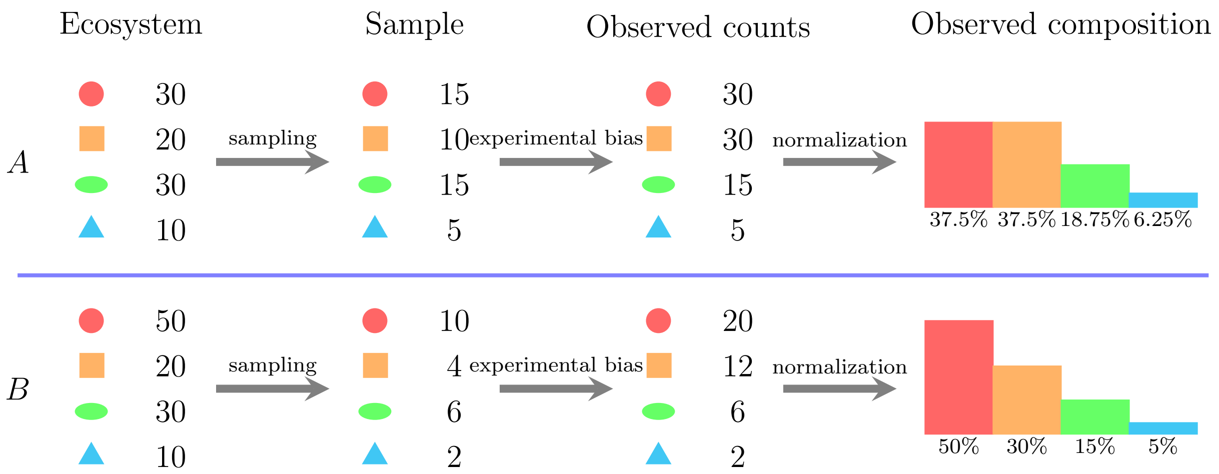

Figure S1: **The limitations inherent in the observed microbial compositional data set.** This figure shows that the sampling process and experimental bias in sequencing workflow create a systemic distinction between absolute abundance in the ecosystem and our observed count data. The observed composition after normalization can also be significantly different from the relative abundance in the original ecosystem owing to the experimental bias. The measurement efficiency factors in this example is $(2, 3, 1, 1)$.

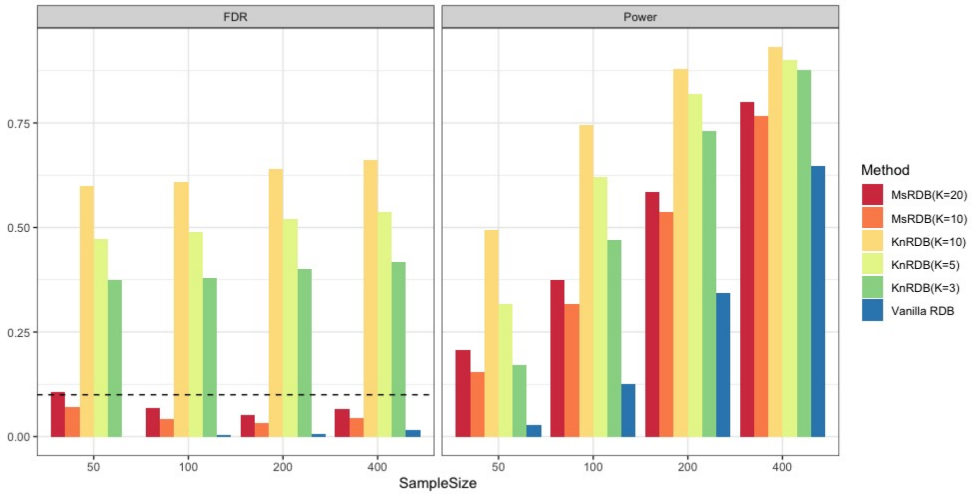

Figure S2: **FDR and power comparisons of different versions of the RDB tests in simulated data sets.** Left and right figures show the FDR and power of various versions of RDB methods when the sample sizes are 50, 100, 200, and 400. The dashed line in the left figure is the target level of FDR at 10%. The results in this figure show that aggregation of information from the neighborhood can increase the power. However, simple aggregation in k-nearest neighbors could result in inflation of false discoveries. In contrast, the MsRDB test increases the power and avoids FDR inflation since only ASVs with similar levels of differential abundance are aggregated.

References

A. Amir, D. McDonald, J. A. Navas-Molina, E. Kopylova, J. T. Morton, Z. Zech X., E. P. Kightley, L. R. Thompson, E. R. Hyde, A. Gonzalez, and R. Knight. Deblur rapidly resolves single-nucleotide community sequence patterns. *MSystems*, 2(2):e00191–16, 2017.

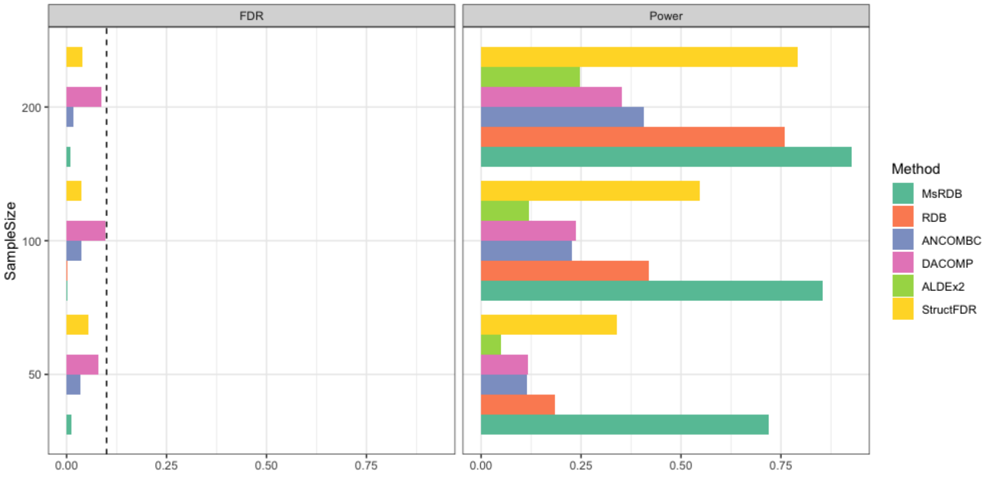

Figure S3: **Comparisons of differential abundance tests in simulated data sets when differential abundant microbes are different genera.** The differential abundant ASVs are all ASVs in genera *Ruminococcus*, *Bacteroides*, *Faecalibacterium*, *Oscillibacter*, and *Blautia*. Left and right figures show the FDR and power of differential abundance tests when the sample sizes are 50, 100, and 200. The dashed line in the left figure is the target level of FDR at 10%. All methods can control FDR at the ASV level very well. The results confirm that aggregating information from the neighborhood can lead to a more powerful test.

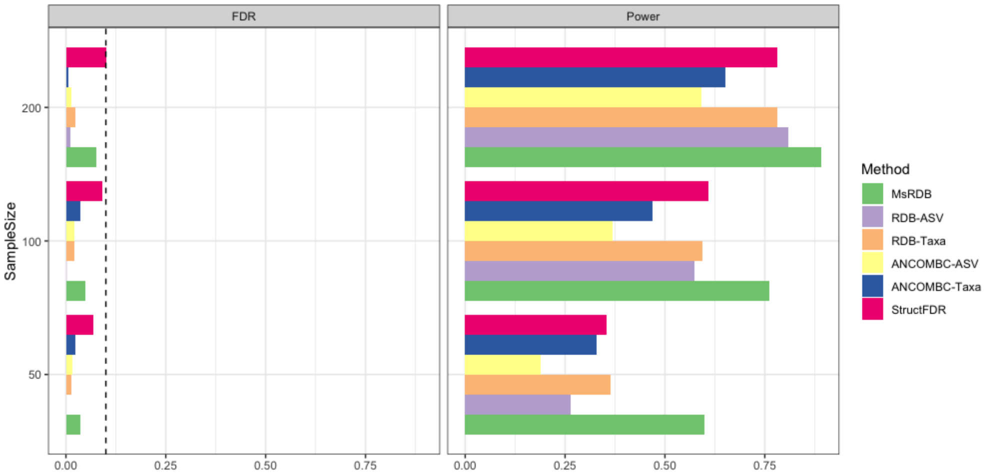

Figure S4: **Comparisons of differential abundance tests in identifying differential abundant genera.** The differential abundant genus is a genus with at least one differential abundant ASV. Left and right figures show the FDR and power of differential abundance tests when the sample sizes are 50, 100, and 200. The dashed line in the left figure is the target level of FDR at 10%. All methods can control FDR at the genus level very well. Choosing genus as analysis units can improve the power of RDB and ANCOM.BC when the sample size is small.

N. A. Bokulich, J. H. Thorngate, P. M. Richardson, and D. A. Mills. Microbial biogeography of wine grapes is conditioned by cultivar, vintage, and climate. *Proceedings of the National Academy of Sciences*, 111(1):E139–E148, 2014.

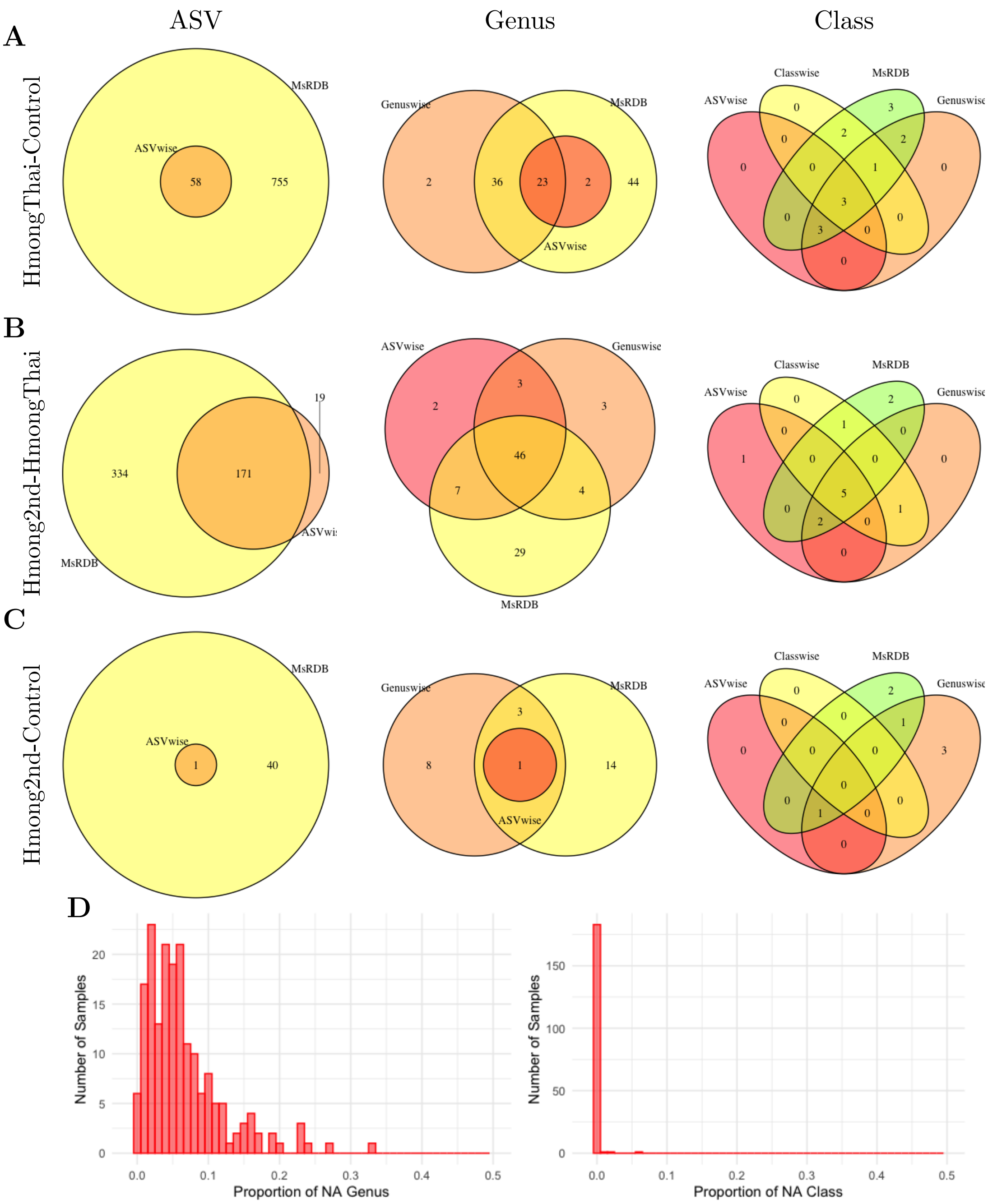

Figure S5: **Comparisons between ASV-wise analysis, taxon-wise analysis, and MsRDB test on the microbiota data set in an immigration study.** (A-C) shows the overlap of results obtained by different analysis strategies in pairwise comparisons of three groups of people. The three rows are the comparisons of HmongThai vs. Control, Hmong2nd vs. HmongThai, and Hmong2nd vs. Control. The first column is the overlap of identified ASV by ASV-wise analysis and the MsRDB test. The second column is the overlap of identified genera by ASV-wise analysis, genus-wise analysis, and the MsRDB test. The third column is the overlap of identified classes by ASV-wise analysis, genus-wise analysis, class-wise analysis, and the MsRDB test. (D) The histograms of proportions of NA genus (left) and class (right) in each sample. Most samples need to remove at least 5% sequences from the genus-wise analysis.

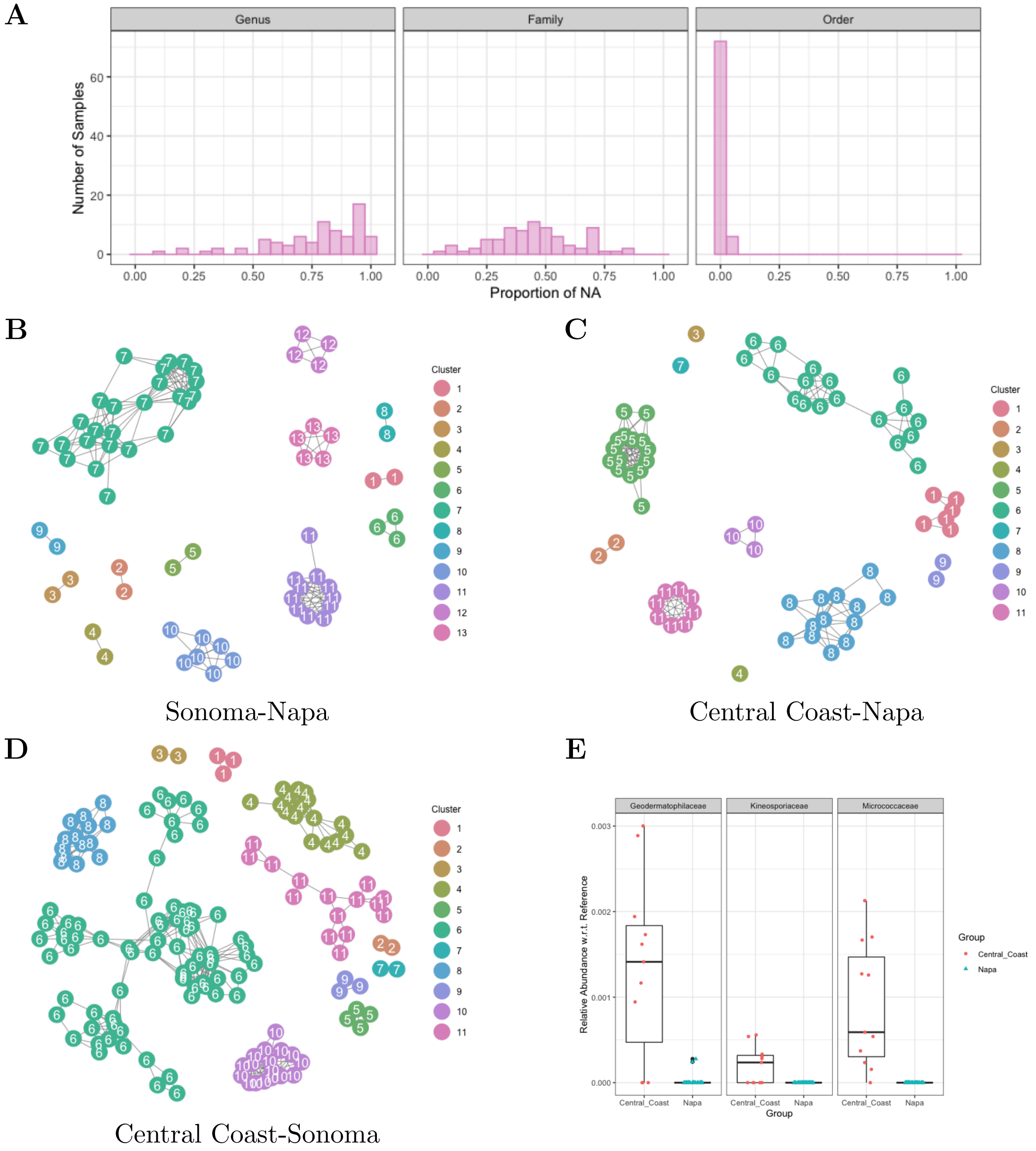

Figure S6: **Analysis of the grape microbiota data set by MsRDB test.** (A) The histograms of proportions of the NA genus (left), class (middle), and order (right) in the grape microbiota data. A considerable amount of sequences are removed from the genus-wise or family-wise analysis. (B-D) The clustering results of significant ASVs in the pairwise comparisons. 13 clusters are obtained in Sonoma vs. Napa comparison, 11 clusters are obtained in Central Coast vs. Napa comparison, and 11 clusters are obtained in Central Coast vs. Sonoma comparison. (E) The box plots show the relative abundance with respect to the reference set in clusters 8, 9, and 10 in Central Coast vs. Napa comparison.

B. Brill, A. Amir, and R. Heller. Testing for differential abundance in compositional counts data, with application to microbiome studies. *The Annals of Applied Statistics*, 16(4): 2648–2671, 2022.

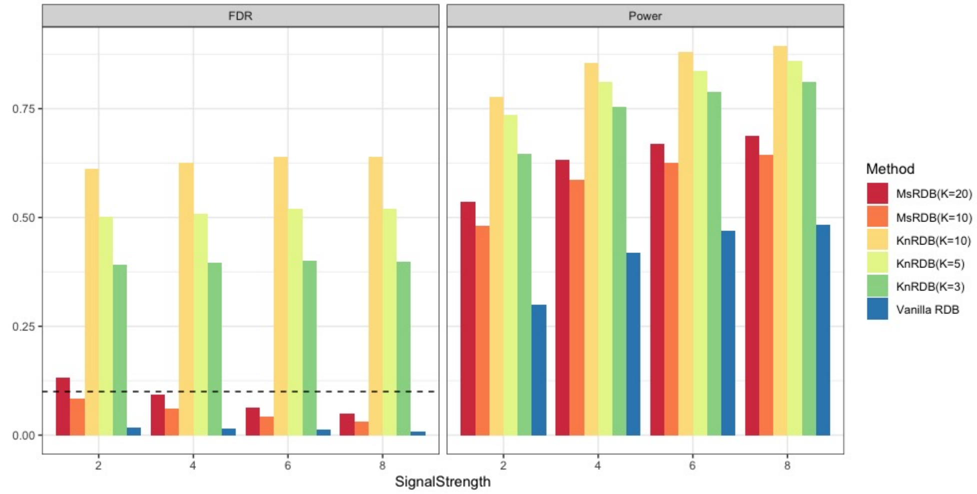

Figure S7: **FDR and power comparisons in simulated data set when the signal strength is different.** Left and right figures show the FDR and power of various versions of RDB methods when the signal strengths are 2, 4, 6, and 8. The dashed line in the left figure is the target level of FDR at 10%. The results in this figure show that the power becomes larger when the signal strength increases.

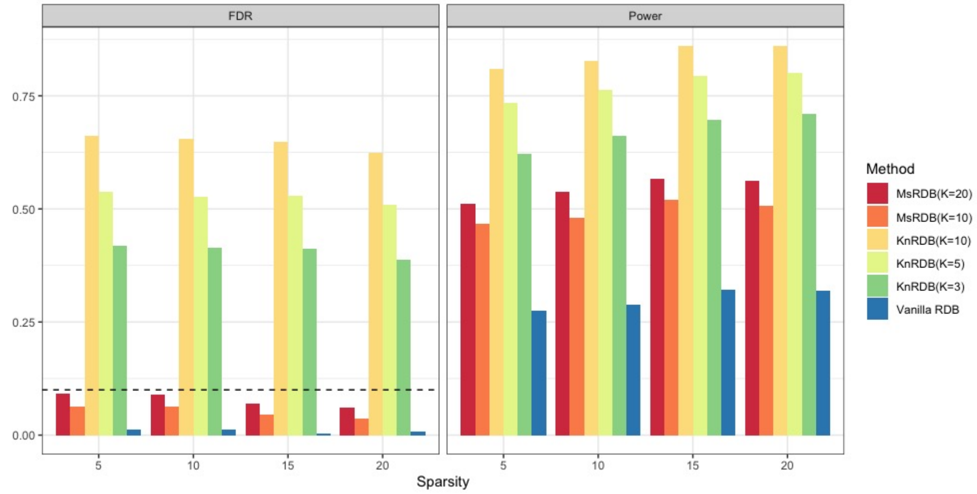

Figure S8: **FDR and power comparisons in simulated data set when the number of differentially abundant ASVs is different.** Left and right figures show the FDR and power of various versions of RDB methods when the numbers of differentially abundant ASVs are 5, 10, 15, and 20. The dashed line in the left figure is the target level of FDR at 10%. The results in this figure show that the performance of various methods is relatively stable when the number of differentially abundant ASVs is different.

J. P. Brooks, D. J. Edwards, M. D. Harwich, M. C. Rivera, J. M. Fettweis, M. G. Serrano, R. A. Reris, N. U. Sheth, B. Huang, P. Girerd, et al. The truth about metagenomics: quantifying and counteracting bias in 16s rrna studies. *BMC microbiology*, 15(1):1–14, 2015.

K. C. G. Chan, S. C. P. Yam, and Z. Zhang. Globally efficient non-parametric inference of average treatment effects by empirical balancing calibration weighting. *Journal of the*

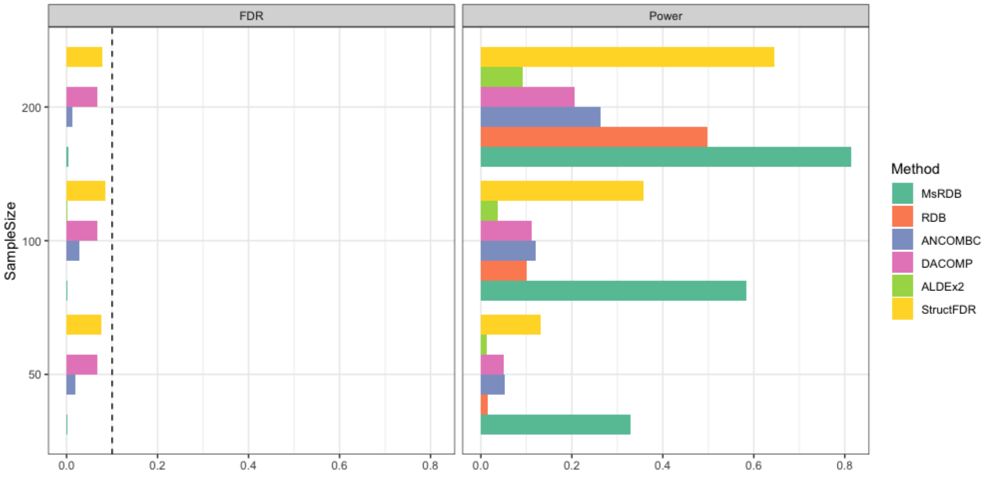

Figure S9: **Comparisons of differential abundance tests in simulated data sets when differential abundant microbes are different classes.** The differential abundant ASVs are all ASVs in classes *Bacteroidia* and *Bacilli*. Left and right figures show the FDR and power of differential abundance tests when the sample sizes are 50, 100, and 200. The dashed line in the left figure is the target level of FDR at 10%. All methods can control FDR at the ASV level very well. The results confirm that aggregating information from the neighborhood can lead to a more powerful test.

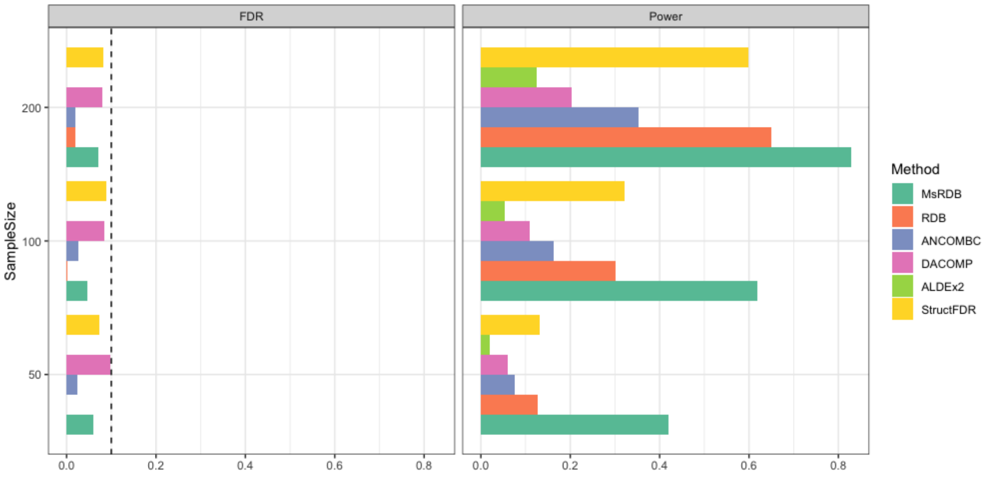

Figure S10: **Comparisons of differential abundance tests in simulated data set when differential abundant microbes are different ASV clusters.** The differential abundant ASVs are ASVs in small ASV clusters. Left and right figures show the FDR and power of differential abundance tests when the sample sizes are 50, 100, and 200. The dashed line in the left figure is the target level of FDR at 10%. All methods can control FDR at the ASV level very well. The results confirm that aggregating information from the neighborhood can lead to a more powerful test.

Royal Statistical Society. Series B, Statistical methodology, 78(3):673, 2016.

G. M. Douglas, V. J. Maffei, J. R. Zaneveld, S. N. Yurgel, J. R. Brown, C. M. Taylor, C. Huttenhower, and M. G. Langille. Picrust2 for prediction of metagenome functions.

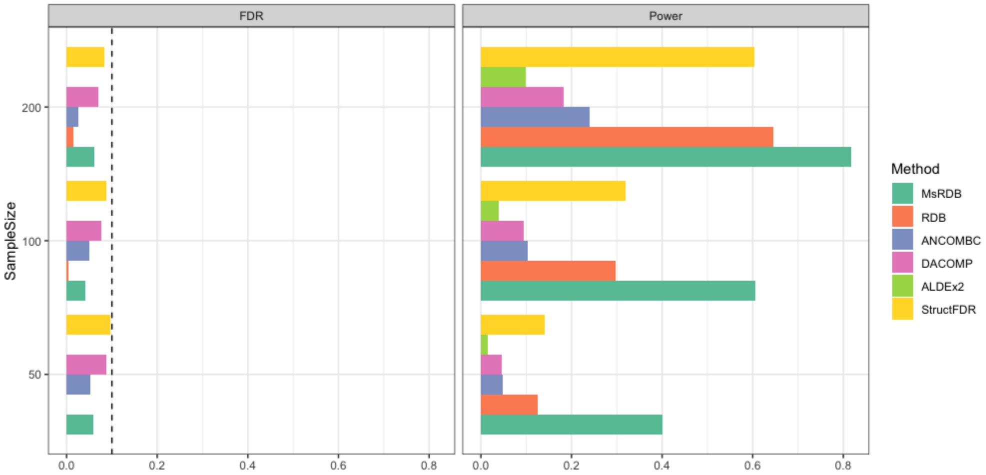

Figure S11: **Comparisons of differential abundance tests in simulated data set when the ASV data has measurement error.** The differential abundant ASVs are ASVs in small ASV clusters, and the ASV data has measurement error. Left and right figures show the FDR and power of differential abundance tests when the sample sizes are 50, 100, and 200. The dashed line in the left figure is the target level of FDR at 10%. All methods are robust to the measurement error.

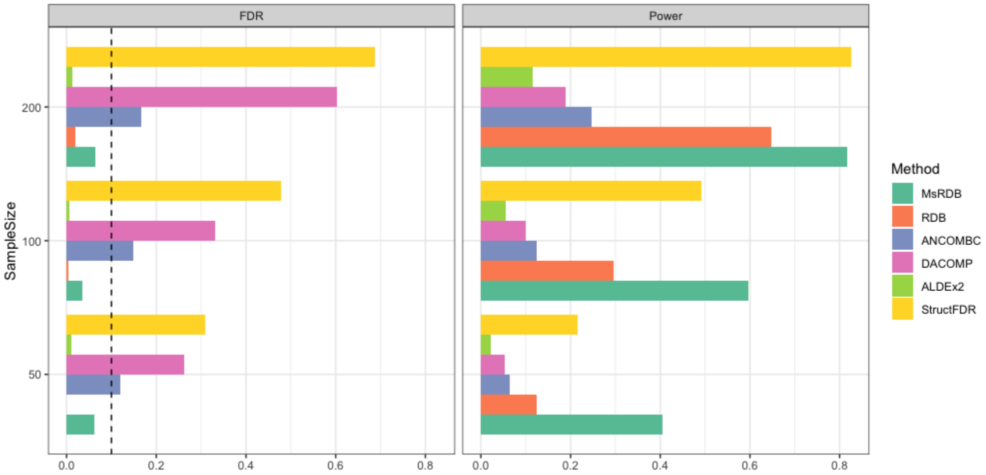

Figure S12: **Comparisons of differential abundance tests in simulated data set when the ASV data has measurement error and unbalanced sequencing depth.** The differential abundant ASVs are ASVs in small ASV clusters, and the ASV data has measurement error and unbalanced sequencing depth. Left and right figures show the FDR and power of differential abundance tests when the sample sizes are 50, 100, and 200. The dashed line in the left figure is the target level of FDR at 10%. Most methods are robust to measurement error and unbalanced sequencing depth. FDR is inflated in DACOMP and StructFDR due to unbalanced sequencing depth.

Nature Biotechnology, 38(6):685–688, 2020.

J. F. Gentleman and R. C. Mullin. The distribution of the frequency of occurrence of nucleotide subsequences, based on their overlap capability. *Biometrics*, pages 35–52, 1989.

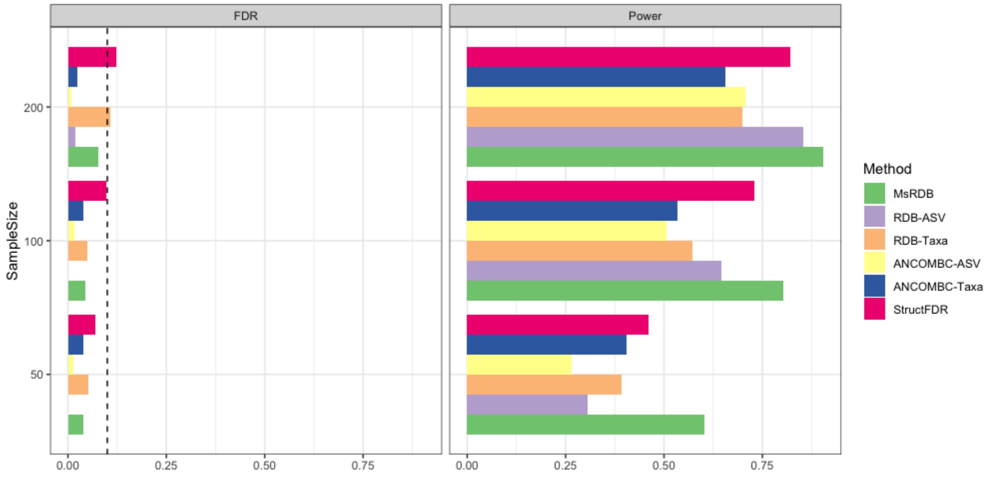

Figure S13: **Comparisons of differential abundance tests in identifying differential abundant families.** The differential abundant family is a family with at least one differential abundant ASV. Left and right figures show the FDR and power of differential abundance tests when the sample sizes are 50, 100, and 200. The dashed line in the left figure is the target level of FDR at 10%. All methods can control FDR at the family level very well. Choosing family as analysis units can improve the power of RDB and ANCOM.BC when the sample size is small.

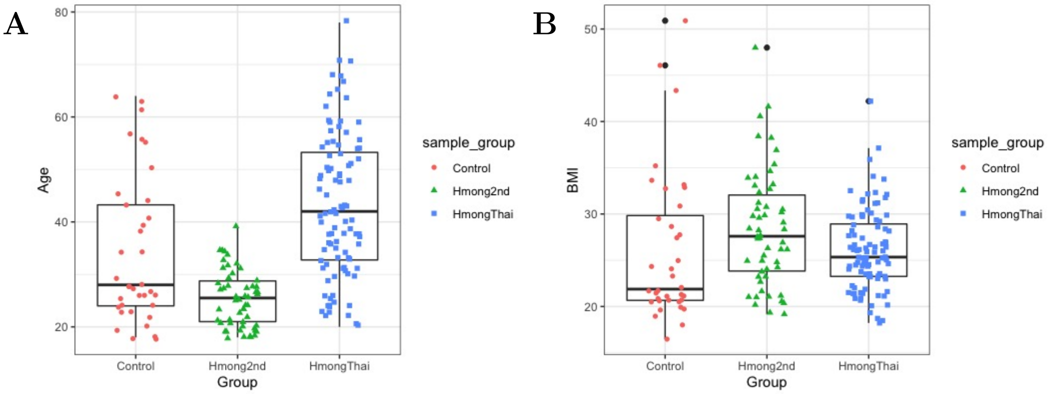

Figure S14: **Degree of covariance balancing in age and BMI in immigration study.** This figure compares the distribution of covariates among HmongThai, Hmong2nd, and Control. Left is age, and right is BMI.

G. W. Imbens and D. B. Rubin. *Causal inference in statistics, social, and biomedical sciences*. Cambridge University Press, 2015.

S. Janssen, D. McDonald, A. Gonzalez, J. A. Navas-Molina, L. Jiang, Z. Z. Xu, K. Winker, D. M. Kado, E. Orwoll, M. Manary, S. Mirarab, and R. Knight. Phylogenetic placement of exact amplicon sequences improves associations with clinical information. *Msystems*, 3 (3):e00021–18, 2018.

Y. Li, H. Zhu, D. Shen, W. Lin, J. H. Gilmore, and J. G. Ibrahim. Multiscale adaptive

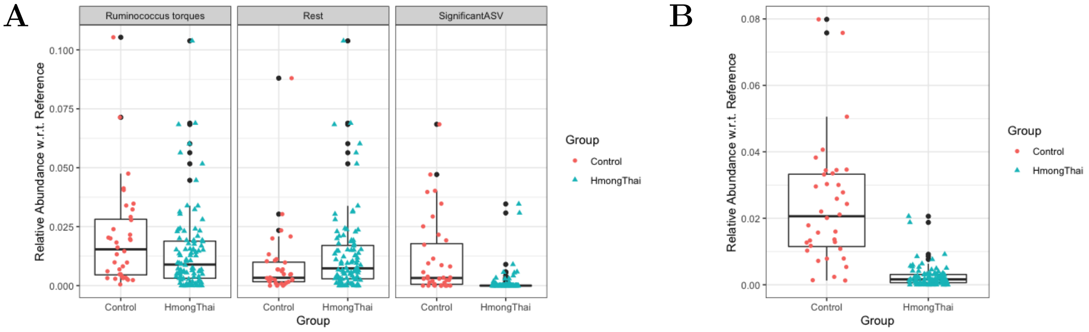

Figure S15: **Taxa detected by MsRDB test on the microbiota data set in immigration study.** The reference set in all box plots is all non-differentially abundant ASVs revealed by the MsRDB test. (A) The box plots show the relative abundance with respect to the reference set in all ASVs assigned to genus *Ruminococcus torques* (left), in significant ASVs within *Ruminococcus torques* (right), and in the rest of ASVs within *Ruminococcus torques* (middle). (B) The box plot shows the relative abundance with respect to the reference set in all significant ASVs assigned to the NA genus.

regression models for neuroimaging data. *Journal of the Royal Statistical Society: Series B*, 73(4):559–578, 2011.

M. R. McLaren, A. D. Willis, and B. J. Callahan. Consistent and correctable bias in metagenomic sequencing experiments. *Elife*, 8:e46923, 2019.

N. D. Olson and J. B. Morrow. Dna extract characterization process for microbial detection methods development and validation. *BMC research notes*, 5:1–14, 2012.

J. Polzehl and V. Spokoiny. Propagation-separation approach for local likelihood estimation. *Probability Theory and Related Fields*, 135(3):335–362, 2006.

J. Polzehl and V. G. Spokoiny. Adaptive weights smoothing with applications to image restoration. *Journal of the Royal Statistical Society: Series B (Statistical Methodology)*, 62(2):335–354, 2000.

M. B. Sohn, R. Du, and L. An. A robust approach for identifying differentially abundant features in metagenomic samples. *Bioinformatics*, 31(14):2269–2275, 2015.

P. Vangay, A. J. Johnson, T. L. Ward, G. A. Al-Ghalith, R. R. Shields-Cutler, B. M. Hillmann, S. K. Lucas, L. K. Beura, E. A. Thompson, L. M. Till, R. Batres, B. Paw, S.L. Pergament, P. Saenyakul, M. Xiong, A. D. Kim, G. Kim, D. Masopust, E. C. Martens, C. Angkurawaranon, R. McGready, P. C. Kashyap, K. A. Culhane-Pera, and D. Knights. Us immigration westernizes the human gut microbiome. *Cell*, 175(4):962–972, 2018.

S. Wang. Robust differential abundance test in compositional data. *Biometrika*, 110(1): 169–185, 2023.

S. Wang, E. T. Arena, J. T. Becker, W. M. Bement, N. M. Sherer, K. W. Eliceiri, and M. Yuan. Spatially adaptive colocalization analysis in dual-color fluorescence microscopy. *IEEE Transactions on Image Processing*, 28(9):4471–4485, 2019.

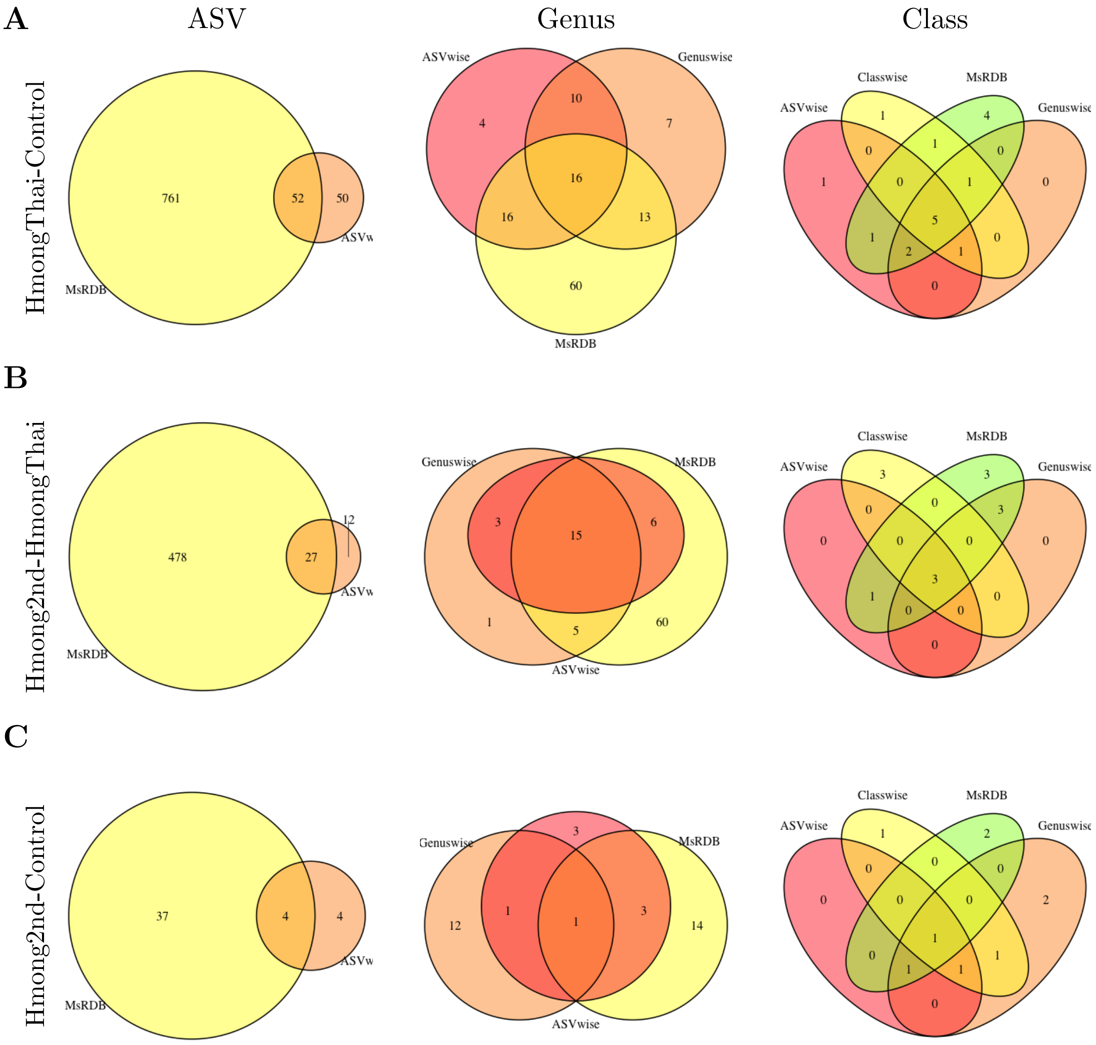

Figure S16: **Comparisons between ASV-wise analysis (ANCOM-BC), taxon-wise analysis (ANCOM-BC), and the MsRDB test on the immigration microbiota data set.** In this figure, ANCOM-BC is applied in ASV-wise analysis and taxon-wise analysis. (A-C) shows the overlap of results obtained by different analysis strategies in pairwise comparisons of three groups of people. The three rows are the comparisons of HmongThai vs. Control, Hmong2nd vs. HmongThai, and Hmong2nd vs. Control.

E. S. Wright. Using decipher v2. 0 to analyze big biological sequence data in r. *R Journal*, 8(1), 2016.

T. Yatsunenko, F. E. Rey, M. J. Manary, I. Trehan, M. G. Dominguez-Bello, M. Contreras, M. Magris, G. Hidalgo, R. N. Baldassano, A. P. Anokhin, A. C. Heath, B. Warner, J. Reeder, J. Kuczynski, J. G. Caporaso, C. A. Lozupone, C. Lauber, J. C. Clemente, D. Knights, R. Knight, and J. I. Gordon. Human gut microbiome viewed across age and geography. *Nature*, 486(7402):222–227, 2012.

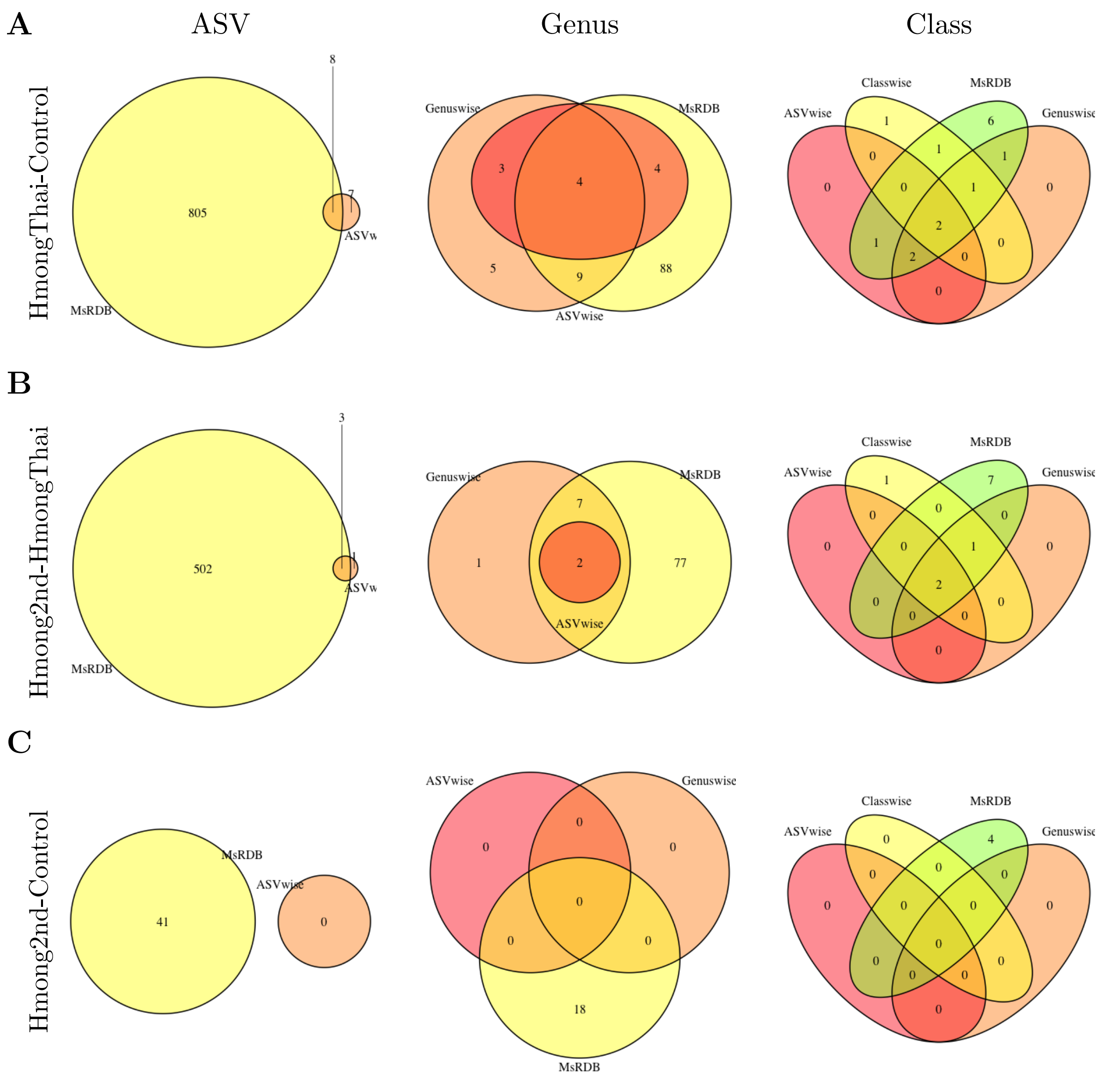

Figure S17: **Comparisons between ASV-wise analysis (ALDEx2), taxon-wise analysis (ALDEx2), and the MsRDB test on the immigration microbiota data set.** In this figure, ALDEx2 is applied in ASV-wise analysis and taxon-wise analysis. (A-C) shows the overlap of results obtained by different analysis strategies in pairwise comparisons of three groups of people. The three rows are the comparisons of HmongThai vs. Control, Hmong2nd vs. HmongThai, and Hmong2nd vs. Control.

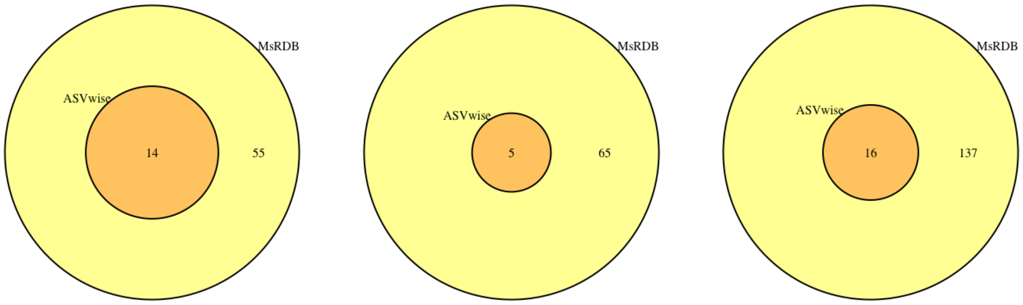

Figure S18: **Comparisons between ASV-wise analysis and MsRDB test on the grape microbiota data set.** This figure shows the overlap of identified ASV by ASV-wise analysis and the MsRDB test. Left is Sonoma vs. Napa, middle is Central Coast vs. Napa, and right is Central Coast vs. Sonoma.
